# Characterization of Uncultivable Bat Influenza Virus Using a Replicative Synthetic Virus

**DOI:** 10.1371/journal.ppat.1004420

**Published:** 2014-10-02

**Authors:** Bin Zhou, Jingjiao Ma, Qinfang Liu, Bhupinder Bawa, Wei Wang, Reed S. Shabman, Michael Duff, Jinhwa Lee, Yuekun Lang, Nan Cao, Abdou Nagy, Xudong Lin, Timothy B. Stockwell, Juergen A. Richt, David E. Wentworth, Wenjun Ma

**Affiliations:** 1 Virology, J. Craig Venter Institute, Rockville, Maryland, United States of America; 2 Department of Diagnostic Medicine/Pathobiology, College of Veterinary Medicine, Kansas State University, Manhattan, Kansas, United States of America; University of Berne, Switzerland

## Abstract

Bats harbor many viruses, which are periodically transmitted to humans resulting in outbreaks of disease (e.g., Ebola, SARS-CoV). Recently, influenza virus-like sequences were identified in bats; however, the viruses could not be cultured. This discovery aroused great interest in understanding the evolutionary history and pandemic potential of bat-influenza. Using synthetic genomics, we were unable to rescue the wild type bat virus, but could rescue a modified bat-influenza virus that had the HA and NA coding regions replaced with those of A/PR/8/1934 (H1N1). This modified bat-influenza virus replicated efficiently *in vitro* and in mice, resulting in severe disease. Additional studies using a bat-influenza virus that had the HA and NA of A/swine/Texas/4199-2/1998 (H3N2) showed that the PR8 HA and NA contributed to the pathogenicity in mice. Unlike other influenza viruses, engineering truncations hypothesized to reduce interferon antagonism into the NS1 protein didn't attenuate bat-influenza. In contrast, substitution of a putative virulence mutation from the bat-influenza PB2 significantly attenuated the virus in mice and introduction of a putative virulence mutation increased its pathogenicity. Mini-genome replication studies and virus reassortment experiments demonstrated that bat-influenza has very limited genetic and protein compatibility with Type A or Type B influenza viruses, yet it readily reassorts with another divergent bat-influenza virus, suggesting that the bat-influenza lineage may represent a new Genus/Species within the *Orthomyxoviridae* family. Collectively, our data indicate that the bat-influenza viruses recently identified are authentic viruses that pose little, if any, pandemic threat to humans; however, they provide new insights into the evolution and basic biology of influenza viruses.

## Author Summary

The identification of influenza virus-like sequences in two different bat species has generated great interest in understanding their biology, ability to mix with other influenza viruses, and their public health threat. Unfortunately, bat-influenza viruses couldn't be cultured from the samples containing the influenza-like nucleic acids. We used synthetic genomics strategies to create wild type bat-influenza, or bat-influenza modified by substituting the surface glycoproteins with those of model influenza A viruses. Although influenza virus-like particles were produced from both synthetic genomes, only the modified bat-influenza viruses could be cultured. The modified bat-influenza viruses replicated efficiently *in vitro* and an H1N1 modified version caused severe disease in mice. Collectively our data show: (1) the two bat-flu genomes identified in other studies are replication competent, suggesting that host cell specificity is the major limitation for propagation of bat-influenza, (2) bat-influenza NS1 antagonizes host interferon response more efficiently than that of a model influenza A virus, (3) bat-influenza has both genetic and protein incompatibility with influenza A or B viruses, and (4) that these bat-influenza lineages pose little pandemic threat.

## Introduction

Bats are present throughout most of the world and account for more than a fifth of mammalian species. They are natural reservoirs of some of the most deadly zoonotic viruses, including rabies virus, Ebola virus, Henipaviruses, and SARS coronavirus [1], [2]. Recently, nucleic acids obtained from bat samples indicated bats may be a reservoir of a new group of influenza viruses (bat-influenza) that are phylogenetically very distantly related to other influenza viruses [3], [4]. Type A, B, and C influenza viruses belong to the *Orthomyxoviridae* family and their genomes are composed of 7–8 negative sense RNA segments (vRNAs). While influenza B (IBV) and C viruses mainly infect human hosts, influenza A virus (IAV) has a broad host range; including humans, marine mammals, horses, pigs, waterfowl, and poultry. New subtypes of IAV, which have novel hemagglutinin (HA) and/or neuraminidase (NA) surface glycoproteins, are introduced into the human population by zoonosis and this periodically leads to devastating pandemics. Past pandemics include the “Spanish flu” (H1N1) in 1918, “Asian flu” (H2N2) in 1957, “Hong Kong flu” (H3N2) in 1968, “Russian flu” (H1N1) in 1977, and the recent “swine origin” flu (pH1N1) in 2009. Pandemic viruses are often reassortant viruses composed of vRNAs that are derived from multiple IAV lineages that previously circulated in swine and/or avian reservoirs (e.g., 1957 avian-human reassortant, 1968 avian-human reassortant, and 2009 avian-swine-human reassortant). The discovery of putative bat-influenza viruses expands the known host species reservoirs that may serve as a source of novel viruses, which is a major concern for public and animal health [4], [5].

Infectious bat-influenza viruses couldn't be isolated [3], [4] and although several structural and biochemical characterization studies have been conducted with the putative bat-influenza HA, NA, and part of PA, none of the vRNAs have been shown to be functional in a replicative virus [3], [4], [6]–[10]. This has led to questions such as: (1) are the putative bat-influenza vRNA sequences identified derived from infectious viruses or are they merely nucleic acid relics harbored in bats [5], (2) are the vRNA segments sequenced from a single bat-influenza virus or are they from multiple potentially incompatible viruses, and (3) were the sequences of the complete gene segments, which is a significant technical challenge, determined accurately. The inability to culture infectious viruses is the major hurdle to confirm the existence of these novel influenza viruses, and to answer important questions, such as pathogenicity in animal models, ability to reassort with other influenza viruses, and their potential risk to public health [5], [11]. The goals of this study were to synthesize the complete viral genome, characterize the bat-influenza virus using non-infectious approaches, then generate a replicative virus, and use it as a model to better understand bat-influenza viruses.

## Results

### Synthetic genomics generated bat-influenza virus-like particles but they were not infectious in many host cell substrates

**Figure 1:**
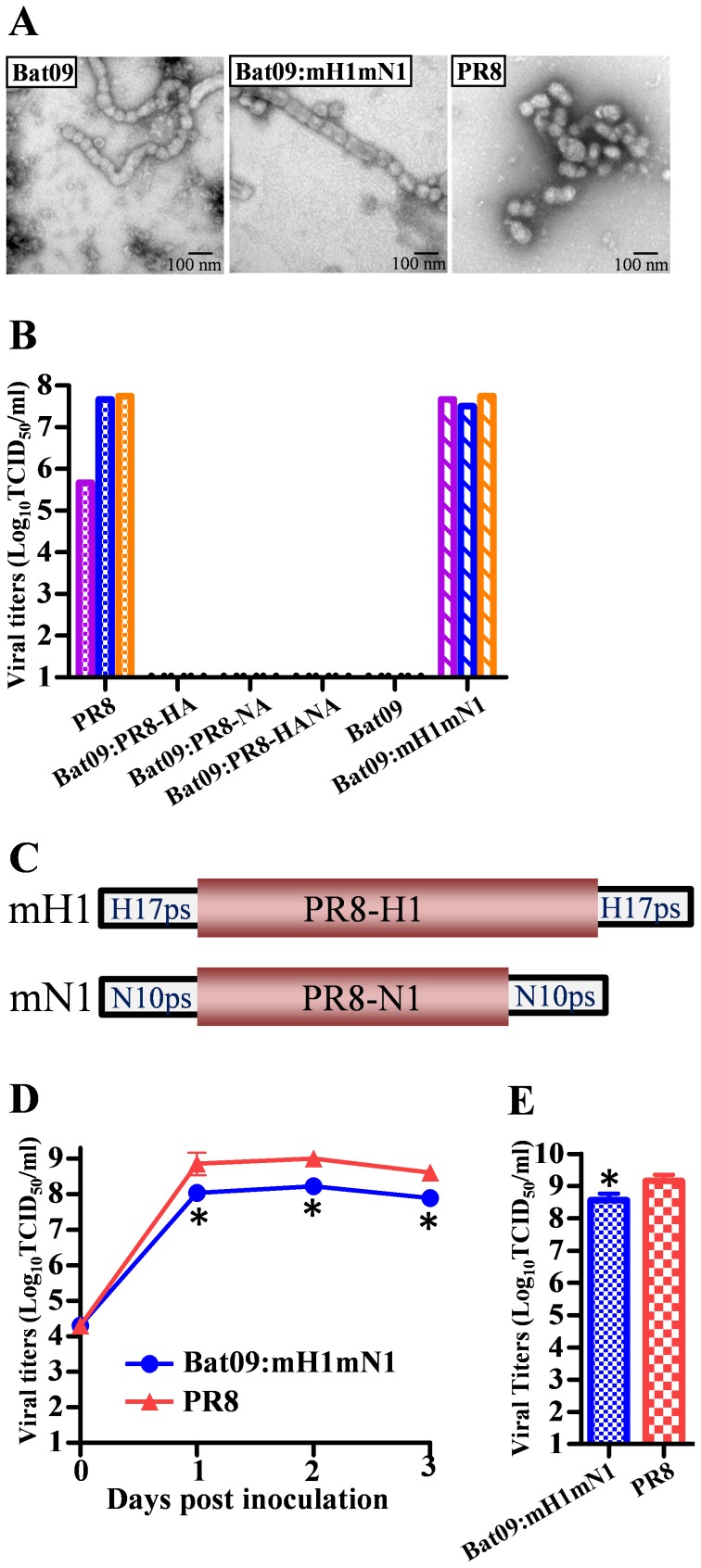
Generation of viruses relevant to Bat09. (**A**) EM picture of Bat09 (left), Bat09:mH1mN1 (middle), and PR8 (right). (**B**) Viral titers in transfection supernatants of Bat09 and PR8-HA/NA reassortants, and Bat09:mH1mN1. Each bar represents an independent rescue experiment. (**C**) The mH1 contains PR8-HA coding region and Bat09-HA packaging region (start codon removed). The mN1 was constructed using the same strategy. (See **Fig. S2A** for details.) (**D**) Bat09:mH1mN1 and PR8 replication kinetics in MDCK cells. (**E**) Peak titers of the viruses in embryonated chicken eggs. *, P<0.05, compared to PR8.

Lack of infectious particles in the original bat specimens is a potential factor in the inability to isolate/culture bat-influenza using multiple host cell substrates [3]. Based on digital sequence information published by Tong *et al.* [3], we synthesized the complete genome of A/little yellow-shouldered bat/Guatemala/164/2009 (H17N10) (**Fig. S1**) and cloned it into reverse genetics plasmids to rescue this putative bat-influenza virus (**Bat09**). Thousands of spherical influenza-like particles budded into the supernatants of human cells (293T) transfected with the Bat09 reverse genetics plasmids (**Fig. 1A**). The supernatants were inoculated into embryonated chicken eggs and cell lines derived from many species (canine (MDCK), mink (Mv1-Lu), swine (ST), African green monkey (Vero), human (A549, Calu-3), and free-tailed bat (Tadarida brasiliensis, Tb1Lu); however, none of the host cell substrates tested supported productive virus infection (determined by serial passage and subsequent real-time RT-PCR).

Previous biochemical and structural studies with purified proteins of Bat09 hemagglutinin (HA) and neuraminidase (NA) indicate that the HA doesn't bind to canonical sialic acid receptors of influenza viruses and the NA doesn't have neuraminidase activity, which is characteristic of IAV and IBV NAs [6]–[9]. To further examine if the HA and NA proteins are the major blocks to the propagation of the Bat09 virus, we attempted to rescue reassortant viruses that contained the 6 internal protein coding vRNAs (PB2, PB1, PA, NP, M, and NS) from Bat09 and the surface glycoprotein vRNAs (HA and/or NA) from a recombinant A/Puerto Rico/8/1934 (PR8). PR8 is a lab adapted H1N1 virus that has been used for many years in research and vaccine settings because it replicates efficiently in embryonated chicken eggs, cell lines (e.g., MDCK) and in the mice, but has low risk to humans. However, the three PR8-HA/NA reassortant genotypes containing the Bat09 internal protein vRNAs couldn't be rescued following transfection (**Fig. 1B**). While the Bat09 internal protein/vRNAs are capable of generating proteins and producing influenza-like particles, they may have critical mutations that were inhibiting infectivity, or they can't cooperate efficiently with the PR8-HA/NA proteins/vRNAs.

### Modified bat-influenza virus could be generated and it replicated efficiently *in vitro, in ovo*, and *in vivo*

To further address the inability to rescue Bat09 or the Bat09:PR8-HA/NA reassortants, we created a modified HA vRNA (mH1) that contained the protein coding region from PR8-H1 flanked by putative cis-acting terminal packaging signals from Bat09 that we hypothesized would be similar to the regions known to be central to packaging of A/WSN/1933 and PR8 [12], [13] (**Fig. 1C** **and Fig. S2**). The Bat09 NA gene segment was modified using a similar strategy to replace the NA coding region with PR8-N1, while the putative bat NA packaging signals were retained (mN1) (**Fig. 1C** **and Fig. S2**). Co-expression of the mH1 and mN1 vRNAs with the six Bat09 internal protein vRNAs efficiently rescued a reassortant Bat09:mH1mN1 virus (**Fig. 1B**). The reassortant Bat09:mH1mN1 formed particles similar to that of Bat09 (**Fig. 1A**) and replicated robustly *in vitro* and *in ovo* (**Fig. 1D, 1E**). Next generation sequencing demonstrated that the consensus sequence of the virus stocks from 1 passage in MDCK cells or embryonated chicken eggs was identical to that of the reverse genetics plasmids. Furthermore, even after 3 passages in MDCK cells, we still didn't identify any nucleotide polymorphisms accounting for >10% of the genomic population that would suggest strong selective pressure on Bat09 genes or the modified HA/NA genes of PR8.

**Figure 2:**
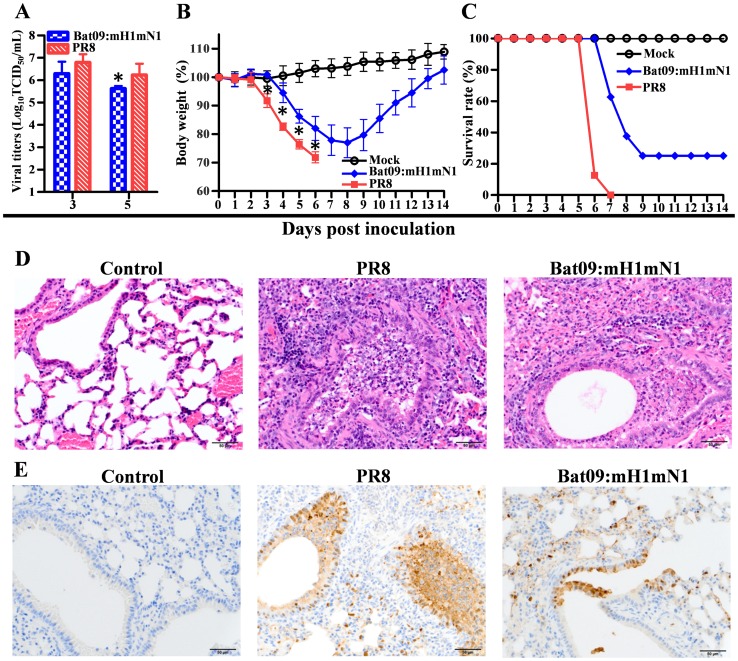
Pathogenicity of Bat09:mH1mN1 and PR8 viruses in mice. (**A**) Virus titers of Bat09:mH1mN1 and PR8 in mouse lungs at 3 and 5 dpi. Each mouse was intranasally inoculated with 10^3^ TCID_50_ of each virus. (**B**) Mouse weight on each day post inoculation was represented as a percentage of the weight on day 0 (100%). Each mouse was intranasally inoculated with 10^4^ TCID_50_ of Bat09:mH1mN1 or PR8. (**C**) Survival rate of the mice inoculated with 10^4^ TCID_50_ of virus. (**D**) H&E staining of microscopic lung sections from mice inoculated with 10^3^ TCID_50_ of virus at 5 dpi. (**E**) IHC staining of lung sections at 5 dpi. *, P<0.05, Bat09:mH1mN1 compared to PR8.

To investigate whether Bat09:mH1mN1 is able to infect and replicate in mice, a mouse study was performed using the mouse adapted PR8 IAV as a positive control. Bat09:mH1mN1 replicated efficiently in mouse lungs (**Fig. 2A**), and caused significant weight loss as early as at 4 days post inoculation (4 dpi) (**Fig. 2B**). The virulence of Bat09:mH1mN1 (75% mortality) was close to that of the **PR8** virus (100% mortality) (**Fig. 2C**). Histopathological analysis showed that the Bat09:mH1mN1 virus caused typical influenza-like lesions characterized by a varying degree of broncho-alveolar epithelial degeneration and necrosis, and interstitial pneumonia. The peribronchiolar and perivascular areas were infiltrated by moderate numbers of lymphocytes and plasma cells (**Fig. 2D**). The histopathology identified correlates with presence of virus antigen in the mouse lungs (**Fig. 2E**).

Next generation sequencing was used to determine if the Bat09 vRNAs were genetically stable in mice. Although nucleotide polymorphisms (at the level of 12%–36%) were detected at sporadic loci throughout the Bat09 vRNAs, each lung sample only had one such polymorphism on average, and none of the mutations were found in more than one mouse. Nonetheless, serial passage of this virus in mice may identify mutations in the Bat09 backbone critical to replication/pathogenesis in mice. We did identify a low level nucleotide polymorphism in the modified PR8 HA at residue at 187 that emerged in multiple Bat09:mH1mN1 inoculated mouse lung samples collected at 3 and 5 dpi (HA-K187E, 10%–20% of the genomic population). This unanticipated result may have also occurred in PR8 inoculated mice; however the lung specimens from these mice were not sequenced.

**Figure 3:**
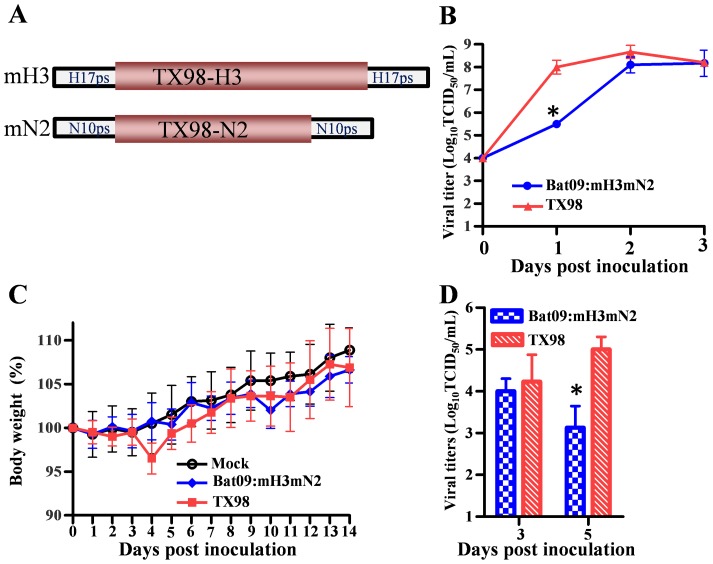
Diagram of the Bat09:mH3mN2 construct, and pathogenicity of Bat09:mH3mN2 and TX98 viruses in mice. (**A**) Modified HA (mH3) and modified NA (mN2). To construct the mH3, TX98-HA coding region was flanked by the putative packaging regions from Bat09-HA and all potential start codons in the Bat09-HA 5′ packaging region were mutated. To construct the mN1, PR8-NA coding region was flanked by the putative packaging regions from Bat09-NA and all start codons in the Bat09-NA 5′ packaging region were mutated. (**B**) Bat09:mH3mN2 and TX98 replication kinetics in MDCK cells. MDCK cells were inoculated at a multiplicity of infection (MOI) of 0.01 TCID_50_/cell with the Bat09:mH3mN2 or TX98 viruses. (**C**) Weight loss of mice mock-infected or infected with Bat09:mH3mN2 or TX98 viruses. Each mouse was intranasally inoculated with 3×10^5^ TCID_50_ of each virus. (**D**) Virus titers of Bat09:mH3mN2 and TX98 viruses in mouse lungs at 3 and 5 dpi. Each mouse was intranasally inoculated with 3×10^4^ TCID_50_ of each virus. *, P<0.05, Bat09:mH3mN2 compared to TX98.

The virulence of the Bat09:mH1mN1 in mice could partly result from the H1 and N1 of the mouse adapted PR8 virus. To further investigate pathogenicity of Bat09-like viruses we rescued another modified Bat09 virus that expresses H3N2 surface glycoproteins from A/swine/Texas/4199-2/1998 (H3N2) (TX98), which we have used in pigs previously [14]. The HA/NA vRNAs of Bat09:mH3mN2 were modified using a similar strategy used to generate the mH1/mN1, whereby the coding regions of Bat09 glycoproteins were replaced with TX98 H3N2, while the putative Bat09 packaging signals were retained (mH3/mN2) (**Fig. 3A**). The rescued Bat09:mH3mN2 virus replicated to peak titers close to that of TX98 (**Fig. 3B**) and both viruses were inoculated into mice to compare the morbidity (weight loss), mortality and virus replication at various times post inoculation. All mice survived infection and both viruses (Bat09:mH3mN2 and TX98) caused little effect on weight gain as compared to the mock inoculated animals (**Fig. 3C**), indicating little overall disease. Titration of virus in the lung tissues showed that the Bat09:mH3mN2 virus replicated as efficiently as the TX98 control in the mice at early time points, yet it appeared to be cleared more rapidly (**Fig. 3D**). This data suggests that some of the pathogenicity observed in the Bat09:mH1mN1 infected mice likely results from the mouse adapted HA/NA of PR8. However, it is clear that the bat influenza internal protein vRNAs do support replication of the modified viruses (Bat09:mH1mN1 and Bat09:mH3mN2) *in vitro*, *in ovo*, and in the mouse lungs. The slightly lower replication efficiency and pathogenicity of those two viruses compared to the corresponding PR8 and TX98 viruses could be ascribable to either the nature of the Bat09 internal protein vRNAs or the engineering of the modified HAs and NAs.

### Bat-influenza NS1 shows strong innate immune suppression in human cells and in mice

**Figure 4:**
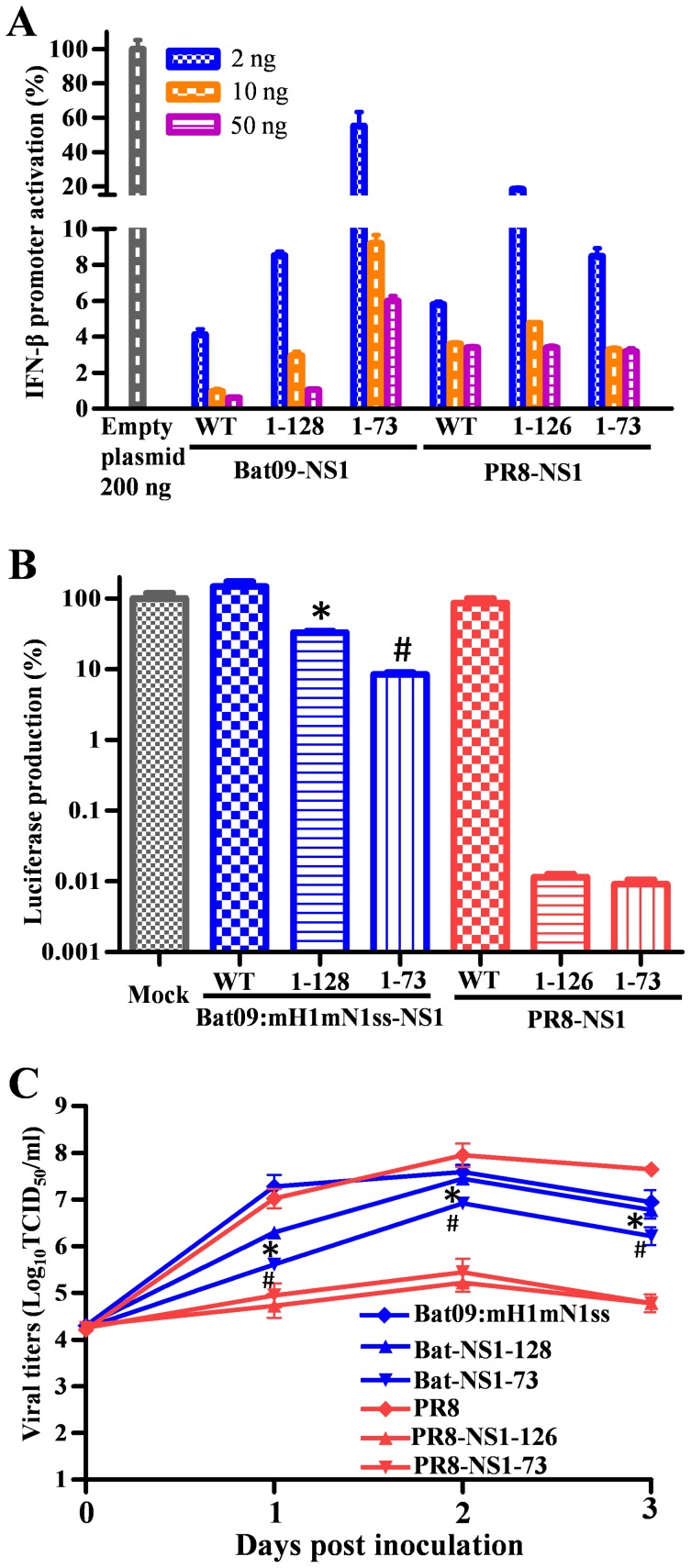
*In vitro* property of Bat-NS1 mutants. (**A**) Luciferase reporter mediated assay to quantitate the NS1 protein inhibition effects on interferon-β promoter activation. (**B**) VSV-luciferase mediated bioassay to quantitate the inhibitory effects on VSV virus infection resulted from the immune response induced by the different NS1 WT or truncated viruses. (**C**) Virus replication kinetics in human lung epithelial Calu-3 cells. * or #, P<0.05, Bat-NS1-128 compared to PR8-NS1-126 (*) and Bat-NS1-73 compared to PR8-NS1-73 (#) are shown in (B) and (C).

Bat-influenza viruses appear to have diverged from IAV a very long time ago and their internal protein vRNAs have many unique features that are not seen in IAVs [3], [4]. Therefore, the biological roles of the various vRNA segments and their protein products are likely to have both similarities and intriguing differences. Many deadly bat viruses (e.g., filoviruses) have evolved powerful molecular mechanisms that inhibit host (e.g., human) immune responses [15]–[18]. Therefore, to gain an understanding of how bat-influenza viruses may evade the host innate immune response we analyzed the Bat09 NS1 protein using interferon induction experiments and carboxy-terminal truncation mutations known to attenuate IAVs. The NS1 protein of IAVs is critical for pathogenicity of many strains because of its ability to antagonize the host interferon response [19]. To compare the direct effect of Bat09-NS1 and PR8-NS1 on interferon-β production, we expressed the proteins ectopically in human HEK-293T and then infected them with Sendai virus to stimulate the innate immune response. Activation of interferon-β promoter was determined by a luciferase mediated reporter assay [16]. Bat09-NS1 inhibited host interferon-β induction comparable to that of the PR8-NS1, and carboxy-terminal truncation of Bat-NS1 protein (NS1-128 and NS1-73, see **Fig. S2C** for diagram) decreased its ability to inhibit interferon-β production (**Fig. 4A**). These results are consistent with the attenuating effect that these NS1 truncations have on PR8 (**Fig. 4A**) and other IAV NS1 proteins; thereby, providing a strategy to generate live attenuated influenza vaccines [14], [20]–[22].

A VSV-luciferase virus mediated bioassay was also performed to compare the effect the NS1 truncations have on the Bat09 viruses' ability to inhibit host innate immune response [22]. The replication of the VSV-luciferase virus, which is sensitive to innate immune activation, is inversely correlated with type I interferon induced by influenza virus. Truncation of the Bat09-NS1 modestly reduced VSV replication, whereas truncation of the PR8-NS1 severely inhibited VSV replication (i.e., luciferase expression) (**Fig. 4B**). These results were confirmed by analysis of influenza virus replication kinetics in a human lung epithelial cell line (**Fig. 4C**). The Bat09-NS1 truncated viruses (**Bat09:mH1mN1ss-NS1-128 and Bat09:mH1mN1ss-NS1-73**) replicated to titers of 10^6^–10^7^ TCID_50_/ml (near wild type NS1; **Bat09:mH1mN1ss**), whereas the PR8-NS1 truncation mutants had 100–1000 fold lower titers than PR8 (**Fig. 4C** **, Fig. S2 for gene and virus diagrams**).

**Figure 5:**
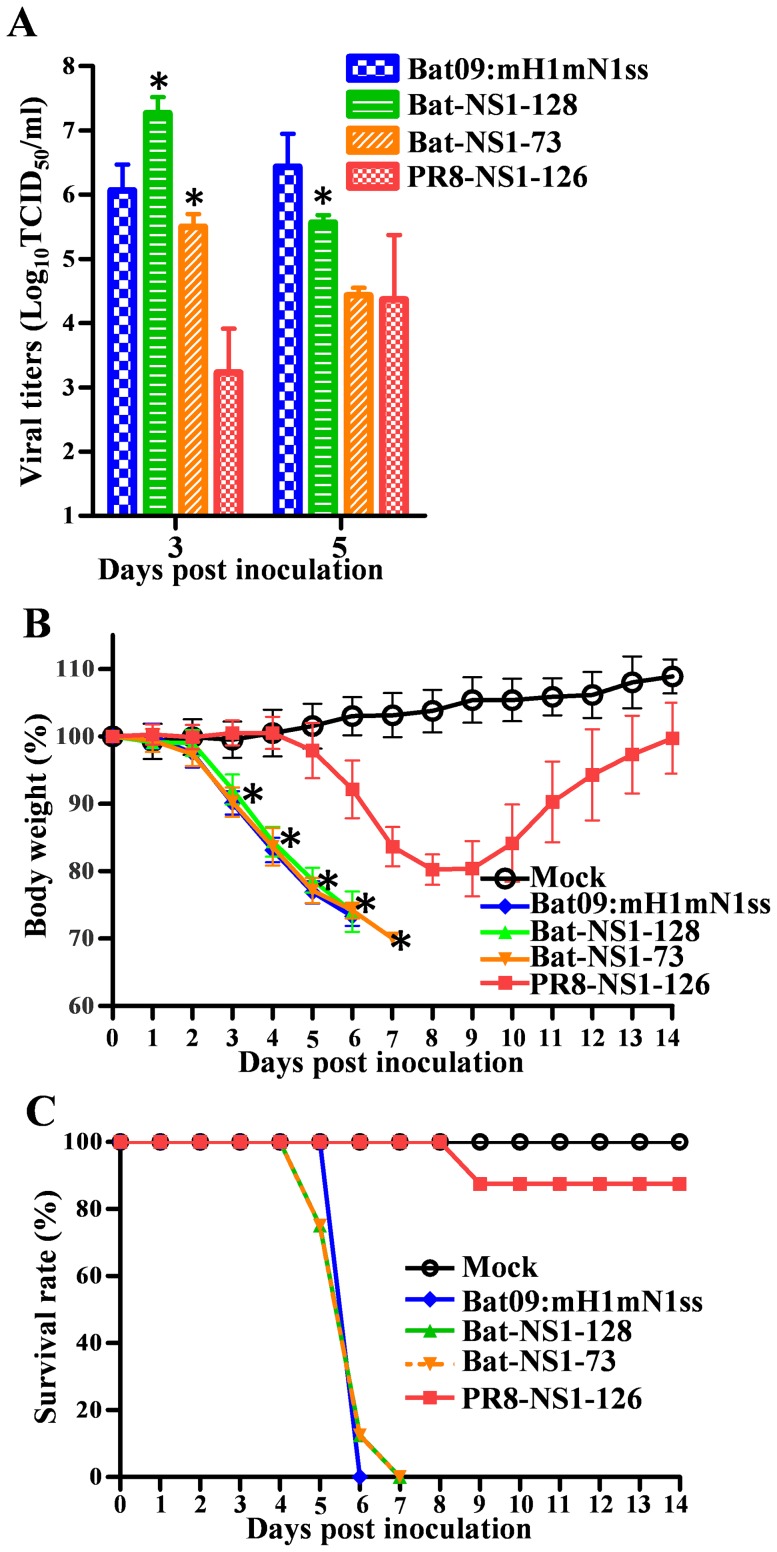
Pathogenicity of Bat-NS1 mutants in mice. (**A**) Virus titers of Bat09:mH1mN1ss and NS1 mutants in mouse lungs at 3 and 5 dpi. Each mouse was intranasally inoculated with 10^4^ TCID_50_ of each virus. (**B**) Mouse weight on each day post inoculation was represented as a percentage of the weight on day 0 (100%). Each mouse was intranasally inoculated with 10^5^ TCID_50_ of the indicated viruses. (**C**) Survival rate of the mice inoculated with 10^5^ TCID_50_ of the viruses. Higher virus doses were used in this experiment based on the PR8-NS1-126 control virus, which caused significant weight loss but had low mortality at 10^5^ TCID_50_ so the attenuation of Bat-NS1 truncated viruses can be appropriately compared to it. *, P<0.05, truncated Bat09:mH1mN1ss-128 and Bat09:mH1mN1ss-73 compared to PR8-NS1-126.

To analyze the impact of these Bat NS1 truncation mutations *in vivo* we inoculated mice with the same panel of modified Bat09 viruses, or the PR8-NS1-126 as a control. In contrast to the significant attenuation conferred by the truncated NS1 in PR8 (**PR8-NS1-126**), recombinant bat-influenza viruses with truncated NS1 genes (**Bat09:mH1mN1ss-NS1-128 and Bat09:mH1mN1ss-NS1-73**) replicated efficiently in the lungs (**Fig. 5A**), caused significant morbidity (**Fig. 5B**), and remained 100% lethal in mice (**Fig. 5C**). Altogether the NS1 studies show that the Bat09 NS1 protein inhibits host interferon-β production and carboxy-terminal truncation mutations reduce its ability to antagonize this response, likely through mechanisms similar to IAV (**Fig. 4A**). However, in contrast to IAV, truncation (NS1-128, NS1-73) of the Bat09 NS1 didn't dramatically impact the viruses' ability to antagonize the host innate response, or replicate and cause disease in mice (**Fig. 4B, C** **and** **Fig. 5**).

### Pathogenesis of the modified Bat09 virus can be manipulated by amino acid substitutions at residues important in virulence of IAVs

**Figure 6:**
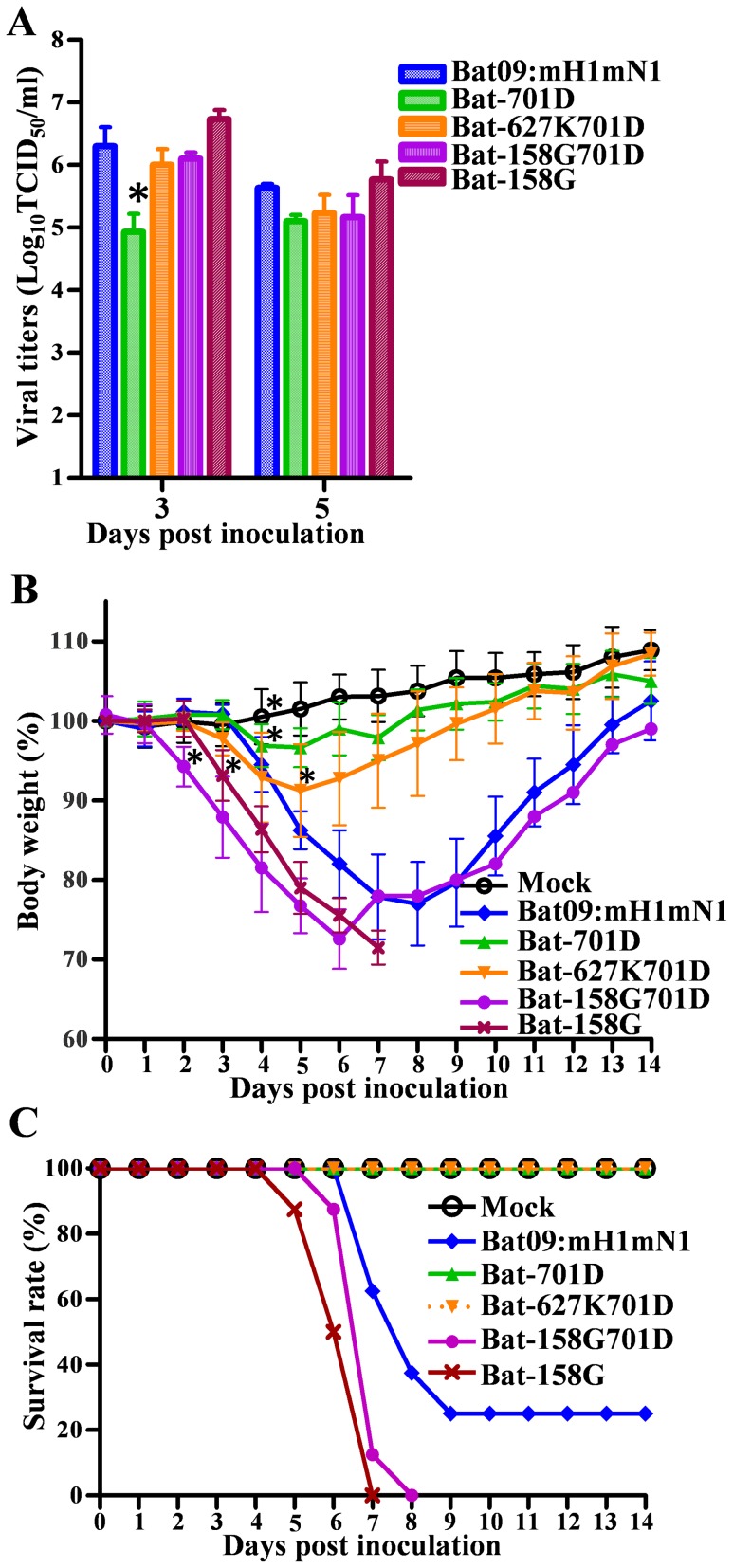
Pathogenicity of Bat-PB2 mutants in mice. (**A**) Virus titers of Bat09:mH1mN1 and PB2 mutants in mouse lungs at 3 and 5 dpi. Each mouse was intranasally inoculated with 10^3^ TCID_50_ of each virus. (**B**) Mouse weight on each day post inoculation was represented as a percentage of the weight on day 0 (100%). Each mouse was intranasally inoculated with 10^4^ TCID_50_ of the indicated viruses. (**C**) Survival rate of the mice inoculated with 10^4^ TCID_50_ of the viruses. *, P<0.05, PB2 mutants compared to Bat09:mH1mN1. For mouse body weight, * is only marked on the first day of each group that is significantly different from Bat09:mH1mN1.

We analyzed the Bat09 PB2 gene because of its central role in the species specificity of IAVs, and some of the critical residues involved are known to be virulence determinants in mice and ferrets [23]–[28]. Asparagine (N) 701 in the PB2 protein is a mammalian-signature in IAVs and when this residue was mutated to aspartic acid (D, an avian-signature) in the modified Bat09 (**Bat-701D**), it decreased virus titers in lungs, morbidity (minor weight loss), and resulted in 100% survival (**Fig. 6**). The bat-influenza PB2 also has a serine (S) residue at position 627, which is unlike either mammalian or avian IAVs. Replacing the serine 627 with the mammalian-signature residue lysine (K) [24], [27] in the context of 701D (**Bat-627K/701D**) increased virus replication in the lungs but only caused slightly more weight loss (compared to the **Bat-701D** virus) and it remained attenuated in mice (**Fig. 6**). In contrast, introducing another virulence marker PB2-E158G [23] into the PB2-N701D virus (**Bat-158G/701D**) dramatically increased the pathogenicity of the Bat09 virus (100% mortality), which was higher than the Bat09 virus with wild type PB2 (**Bat09:mH1mN1**, **Fig. 6**). In addition, introducing the PB2-E158G (**Bat-158G**) into the wild type PB2 resulted the most significant increase of virus replication, morbidity, and mortality (**Fig. 6**), indicating there is an additive effect between the two virulence determinants (PB2-158G and PB2-701N) in the Bat09 PB2. All viruses collected from mouse lungs were deep sequenced to confirm the stability of the engineered mutations and although sporadic nucleotide polymorphisms (10% - 44%) were detected in the viral genomes (1 to 2 such polymorphisms per mouse sample on average), none of them occurred at the engineered loci. The high genetic stability of the modified Bat09 viruses in mice is consistent with the notion that the bat influenza viruses are mammalian viruses that have been evolving and adapting in the bats for a long period of time.

**Figure 7:**
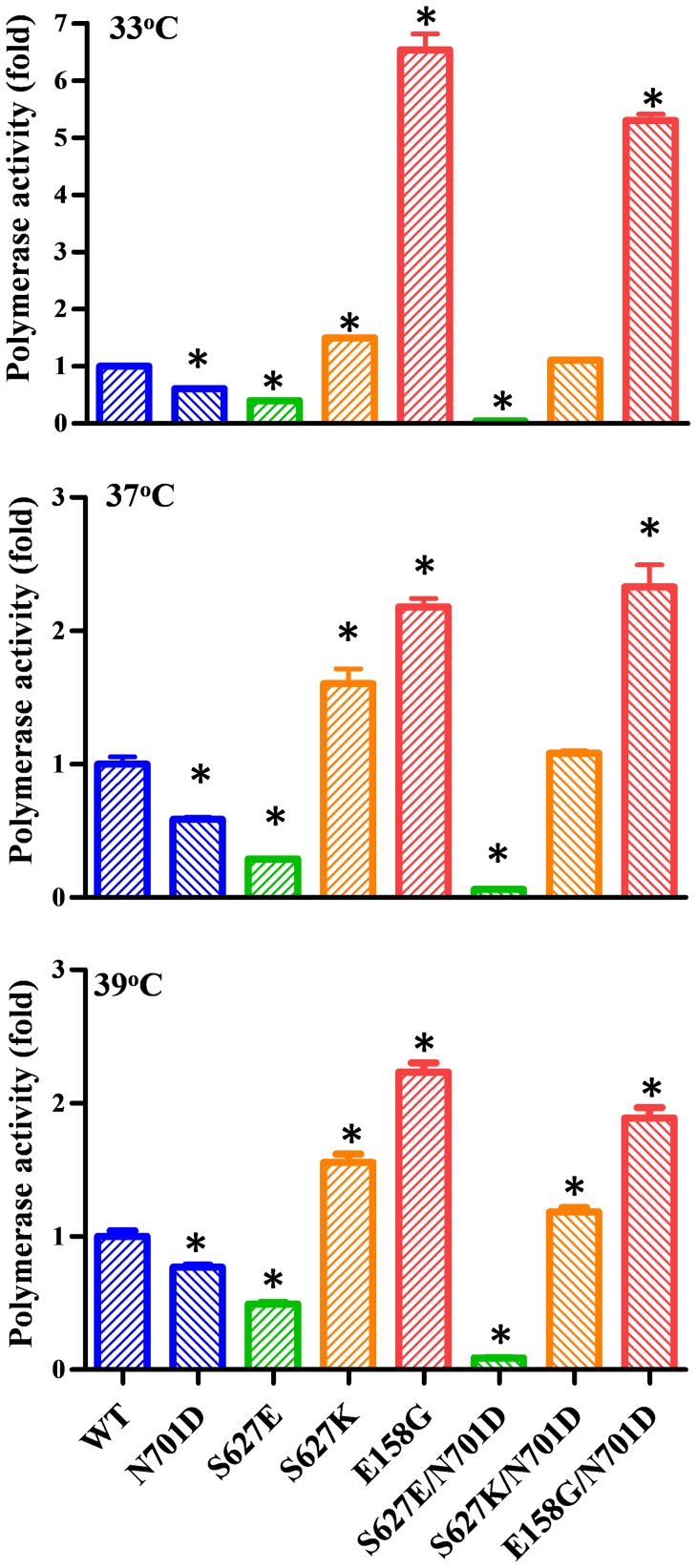
Polymerase activity of Bat09 with wild type and mutant PB2. A luciferase mediated mini-genome replication assay was performed at 33°C, 37°C, and 39°C by co-transfecting 293T cells with Bat09 PB2 (WT or mutant), PB1, PA, NP, and a vRNA-like luciferase reporter. Relative luciferase activity were determined to represent the viral polymerase activity. *, P<0.05, compared to WT.

To determine the molecular basis for the altered pathogenicity imparted by the various mutations in the PB2 we examined their effects on the viral polymerase activity in human 293T cells using a luciferase-mediated mini-genome replication assay (**Fig. 7**). At all temperatures tested, the PB2-N701D mutation decreased the polymerase activity and the PB2-E158G mutation enhanced the polymerase activity, consistent with the decreased and increased pathogenicity in mice, respectively (**Fig. 6**). Interestingly, the PB2-627S showed intermediate polymerase activity compared to the PB2-627K and PB2-627E (**Fig. 7**). In addition, the polymerase activity of the PB2-158G and PB2-627E/K mutants decreased proportionally when they were combined with the PB2-701D mutation (**Fig. 7**). This result is consistent with the observation that Bat-158G/701D appeared to be less pathogenic than the Bat-158G virus (**Fig. 6**). Collectively, the data collected on the Bat09 PB2 show that amino acid residues known to be important in replication, species specificity, transmission, and/or pathogenesis of IAV are important in the replication and pathogenesis of Bat09.

### Internal protein-coding vRNAs of bat-influenza don't efficiently reassort with IAV or IBV

**Figure 8:**
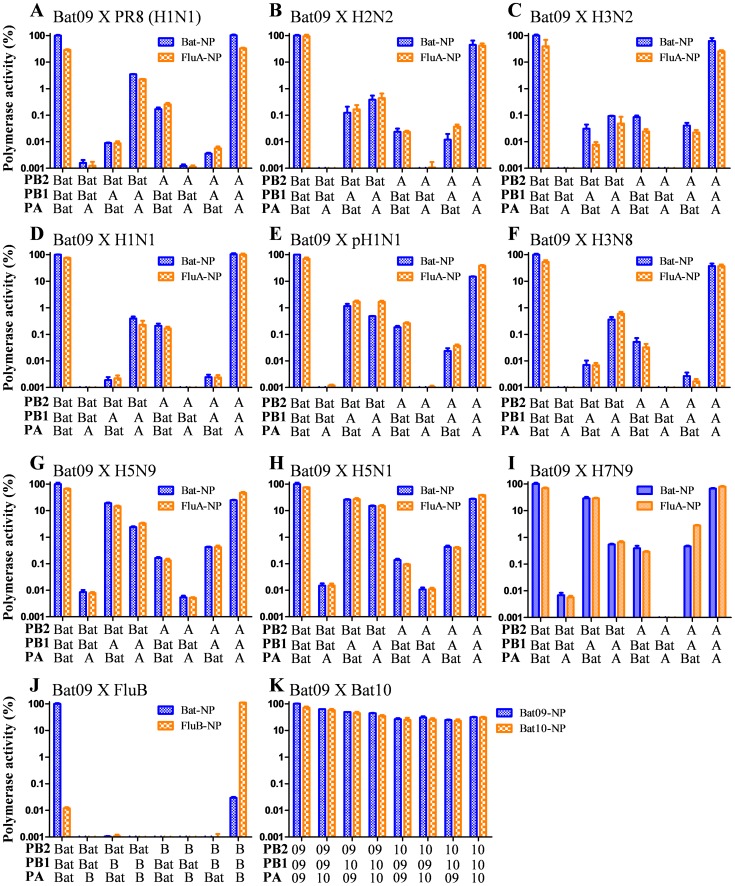
Compatibility of the PB2, PB1, PA and NP originated from Bat09 and other influenza viruses. A mini-genome replication assay was used to determine the compatibility of the different RdRp components. (**A–I**) PB2, PB1, PA, and NP from Bat09 and various influenza A viruses as indicated. (**J**) PB2, PB1, PA and NP from Bat09 and B/Russia/69. (**K**) PB2, PB1, PA and NP from Bat09 and Bat10. The vRNA reporters used for the compatibility test between Bat09 and IAVs (Fig. 8A–I) were an equal ratio of pPolI-Bat-NS-Luc and pPolI-FluA-NS-Luc. For compatibility test between Bat09 and IBV (Fig. 8J) the vRNA reporters used were pPolI-Bat-NS-Luc and pPolI-FluB-NS-Luc. For compatibility test between Bat09 and Bat10 (Fig. 8K) only the pPolI-Bat-NS-Luc plasmid was used.

Reassortment of IAVs is important in the evolution of IAVs and generation of panzootic and pandemic strains. Furthermore, efficient replication of bat-influenza internal protein vRNAs in human cells and mice, as well as their pathogenicity, necessitated an assessment of reassortment potential between Bat09 and other influenza viruses. Replication of vRNAs from different parental viruses is a factor critical in the generation of reassortant progeny. Transcription/replication of mini-genome reporter constructs showed that the viral RNA dependent RNA polymerase (RdRp), which is a heterotrimer of PB1, PB2, and PA, from bat-influenza, IAVs, and IBVs generally recognize and transcribe their cognate vRNAs more efficiently than non-cognate vRNAs (**Fig. S3**). Intriguingly, the Bat09 polymerase replicated the IBV reporter very efficiently (**Fig. S3**). Additionally, most RdRp combinations (PB2, PB1, PA) between bat-influenza and IAVs nearly abolished the polymerase activity in this very sensitive mini-genome reporter assay (**Fig. 8A–I**). Interestingly, the NP protein, which is a single-strand RNA-binding nucleoprotein, is completely compatible between Bat09 and IAVs (**Fig. 8A–I**), but it is incompatible between the bat-influenza and IBV (**Fig. 8J**).

Although some gene segment combinations showed limited polymerase activity in the mini-genome assays, we couldn't generate any reassortant viruses using reverse genetics between Bat09:mH1mN1 and PR8 that contain partly compatible RdRp components (e.g., Bat-PB2/PR8-PB1/PR8-PA), including the highly compatible NP vRNA/protein (**Table 1** and **Table 2**). Instead, the PR8-M segment could unidirectionally substitute for the Bat09-M segment (**Table 2**). This likely results from the highly conserved nature of the M vRNA and proteins (M1, M2). Swapping the putative cis-acting packaging signals of the Bat-NP and known packaging signals of the PR8-NP, or between the Bat-NS and PR8-NS didn't enable rescue of viruses containing either the NP or NS vRNAs in a heterologous virus background (**Table 3** **and see Fig. S2 for diagrams**).

**Table 1:** Rescue efficiency of PB2, PB1, PA reassortants between Bat09:mH1mN1 and PR8.

	PB2	PB1	PA	NP,M,NS	HA,NA	Rescue*
**1**	Bat	Bat	Bat	Bat	mH1,mN1	**++++**
**2**	PR8	Bat	Bat	Bat	mH1,mN1	Neg
**3**	Bat	PR8	Bat	Bat	mH1,mN1	Neg
**4**	Bat	Bat	PR8	Bat	mH1,mN1	Neg
**5**	PR8	PR8	Bat	Bat	mH1,mN1	Neg
**6**	PR8	Bat	PR8	Bat	mH1,mN1	Neg
**7**	Bat	PR8	PR8	Bat	mH1,mN1	Neg
**8**	PR8	PR8	PR8	Bat	mH1,mN1	Neg
**9**	Bat	Bat	Bat	PR8	PR8	Neg
**10**	PR8	Bat	Bat	PR8	PR8	Neg
**11**	Bat	PR8	Bat	PR8	PR8	Neg
**12**	Bat	Bat	PR8	PR8	PR8	Neg
**13**	PR8	PR8	Bat	PR8	PR8	Neg
**14**	PR8	Bat	PR8	PR8	PR8	Neg
**15**	Bat	PR8	PR8	PR8	PR8	Neg
**16**	PR8	PR8	PR8	PR8	PR8	**++++**

*Rescue efficiency definition.

**Very easy (++++)**: P0 viral titer 10^6^–10^8^ TCID_50_/ml, or severe CPE observed in P1 within 1 dpi;

**Moderate (+++)**: P0 titer 10^4^–10^6^ TCID_50_/ml, or obvious CPE observed in P1 within 2 dpi;

**Difficult (++)**: P0 titer 10^2^–10^4^ TCID_50_/ml, or weak CPE observed in P1 within 4 dpi;

**Very difficult (+)**: P0 titer lower than 10^2^ TCID_50_/ml, or CPE not observed until P2/P3;

**Negative (Neg)**: rescue failed, no CPE observed through passage 3.

For each combination, the rescue was repeated at least 3 times.

**Table 2:** Rescue efficiency of internal gene reassortants between Bat09:mH1mN1 and PR8.

	Pols*	NP	M	NS	HA,NA	Rescue**
**1**	Bat	Bat	Bat	Bat	mH1,mN1	**++++**
**2**	Bat	Bat	Bat	PR8	mH1,mN1	Neg
**3**	Bat	Bat	PR8	Bat	mH1,mN1	**+++**
**4**	Bat	Bat	PR8	PR8	mH1,mN1	Neg
**5**	Bat	PR8	Bat	Bat	mH1,mN1	Neg
**6**	Bat	PR8	Bat	PR8	mH1,mN1	Neg
**7**	Bat	PR8	PR8	Bat	mH1,mN1	Neg
**8**	Bat	PR8	PR8	PR8	mH1,mN1	Neg
**9**	PR8	Bat	Bat	Bat	mH1,mN1	Neg
**10**	PR8	Bat	Bat	PR8	mH1,mN1	Neg
**11**	PR8	Bat	PR8	Bat	mH1,mN1	Neg
**12**	PR8	Bat	PR8	PR8	mH1,mN1	Neg
**13**	PR8	PR8	Bat	Bat	mH1,mN1	Neg
**14**	PR8	PR8	Bat	PR8	mH1,mN1	Neg
**15**	PR8	PR8	PR8	Bat	mH1,mN1	Neg
**16**	PR8	PR8	PR8	PR8	mH1,mN1	Neg
**17**	Bat	Bat	Bat	Bat	PR8	Neg
**18**	Bat	Bat	Bat	PR8	PR8	Neg
**19**	Bat	Bat	PR8	Bat	PR8	Neg
**20**	Bat	Bat	PR8	PR8	PR8	Neg
**21**	Bat	PR8	Bat	Bat	PR8	Neg
**22**	Bat	PR8	Bat	PR8	PR8	Neg
**23**	Bat	PR8	PR8	Bat	PR8	Neg
**24**	Bat	PR8	PR8	PR8	PR8	Neg
**25**	PR8	Bat	Bat	Bat	PR8	Neg
**26**	PR8	Bat	Bat	PR8	PR8	Neg
**27**	PR8	Bat	PR8	Bat	PR8	Neg
**28**	PR8	Bat	PR8	PR8	PR8	Neg
**29**	PR8	PR8	Bat	Bat	PR8	Neg
**30**	PR8	PR8	Bat	PR8	PR8	Neg
**31**	PR8	PR8	PR8	Bat	PR8	Neg
**32**	PR8	PR8	PR8	PR8	PR8	**++++**

*Pols = Co-transfection of PB1, PB2, and PA reverse genetics plasmids.

**Rescue efficiency defined in Table 1. For each combination, the rescue was repeated at least 3 times.

**Table 3:** Rescue efficiency of reassortants with NP and NS containing modified packaging signals.

	PB2	PB1	PA	NP	M	NS	HA,NA	Rescue*
**1**	Bat	Bat	Bat	Bat	Bat	Bat	mH1,mN1	**++++**
**2**	Bat	Bat	Bat	Batps-PR8-NP	Bat	Bat	mH1,mN1	Neg
**3**	Bat	Bat	Bat	Bat	Bat	Batps-PR8-NS	mH1,mN1	Neg
**4**	Bat	Bat	Bat	Batps-PR8-NP	Bat	Batps-PR8-NS	mH1,mN1	Neg
**5**	Bat	Bat	Bat	PR8ps-Bat-NP	Bat	Bat	mH1,mN1	Neg
**6**	Bat	Bat	Bat	Bat	Bat	PR8ps-Bat-NS	mH1,mN1	**+**
**7**	PR8	PR8	PR8	PR8ps-Bat-NP	PR8	PR8	PR8	Neg
**8**	PR8	PR8	PR8	PR8	PR8	PR8ps-Bat-NS	PR8	Neg
**9**	PR8	PR8	PR8	PR8ps-Bat-NP	PR8	PR8ps-Bat-NS	PR8	Neg
**10**	PR8	PR8	PR8	Batps-PR8-NP	PR8	PR8	PR8	Neg
**11**	PR8	PR8	PR8	PR8	PR8	Batps-PR8-NS	PR8	**+++**
**12**	PR8	PR8	PR8	PR8	PR8	PR8	PR8	**++++**

*Rescue efficiency defined in Table 1.

For each combination, the rescue was repeated at least 3 times.

### HA and NA vRNAs of bat and other influenza viruses have limited compatibility

Low efficiency of packaging at least some vRNA segments from the heterologous virus is also a major restrictive factor for reassortment. For instance, a reassortant virus containing six internal protein vRNAs from Bat09 and the HA and NA from PR8 couldn't be rescued, whereas the PR8 HA and NA coding regions flanked by Bat09 packaging regions (mH1 and mN1) can efficiently reassort with the Bat09 internal genes (**Fig. 1B** **and** **Table 4**). Nevertheless, PR8 HA and NA can individually reassort (7∶1) with the Bat09 six internal protein vRNAs when mN1 or mH1 were provided, respectively (**Table 4**). The inability to rescue the 6∶2 reassortant Bat09:PR8-H1N1 virus may result from compounding the low efficiency of packaging for each of the wild type PR8-HA and PR8-NA vRNAs into the bat-influenza backbone.

**Table 4:** Rescue efficiency of reassortants containing HA and NA packaging single from other viruses.

	Internal	HA	NA	Rescue*
**1**	Bat	mH1	mN1	**++++**
**2**	Bat	PR8	mN1	**++**
**3**	Bat	mH1	PR8	**+**
**4**	Bat	PR8	PR8	Neg
**5**	PR8	PR8	PR8	**++++**
**6**	PR8	mH1	PR8	Neg
**7**	PR8	PR8	mN1	**+++**
**8**	PR8	mH1	mN1	Neg
**9**	PR8	mH1ss	PR8	Neg
**10**	PR8	PR8	mN1ss	**+++**
**11**	PR8	mH1ss	mN1ss	Neg
**12**	PR8	PR8	Batps/B-NA	**+++**

*Rescue efficiency definition described in Table 1.

The mN1 can also reassort with the other seven segments from PR8, even when many silent substitutions (ss) were introduced into the N1 coding regions to disrupt the remaining PR8 packaging signals (**Table 4**). Actually, another modified NA that contains the coding region from IBV NA flanked by the putative packaging region of the Bat09-NA (Bat-N10ps-FluB-NA) can also be rescued in the PR8 background, strongly suggesting that the bat-influenza NA segment could be efficiently packaged into the PR8 virus, whereas the Bat09 HA packaging signal didn't mediate efficient packaging of the mH1 into the PR8 backbone (**Table 4**).

### Interrogation of reassortment between IAV and modified Bat09 using a classical co-infection approach

While the generation of reassortants through plasmid-based reverse genetics is a powerful and sensitive way to rescue influenza viruses, it's difficult to generate every possible gene constellation and accompanying minor nucleotide variations that could give rise to progeny reassortants during co-infection. Therefore, we attempted to generate reassortants between a modified Bat09 virus and PR8 using a classical co-infection approach. However, when MDCK cells were inoculated at a high multiplicity of infection (MOI) with both PR8 and Bat09:mH1mN1 viruses, reassortment between the two parental viruses was not detected. We plaque purified 118 progeny viruses from the co-infection and 53 of them were the parental PR8 virus and 65 of them were the parental Bat09:mH1mN1 virus. Although more exhaustive classical reassortant studies are needed to completely evaluate the generation of natural reassortants between these viruses, the data indicate that PR8 and Bat09:mH1mN1 don't efficiently reassort.

### Divergent bat-influenza viruses are highly compatible for reassortment

Recently, another bat-influenza virus A/flat-faced bat/Peru/033/2010 (H18N11) (**Bat10**) was identified in Peru and phylogenetic analysis indicated this virus diverged from the bat-influenza viruses identified in Guatemala (e.g., Bat09) so long ago that genetic diversity between these two bat-influenza viruses is higher than that of IAVs [4]. Reassortment of the PB2, PB1, PA, and NP segments in mini-genome polymerase activity assay demonstrated that the Bat09 and Bat10 viruses were fully compatible (**Fig. 8K**). Most importantly, successful reassortment between the two modified bat viruses (Bat09:mH1mN1ss and Bat10:mH1mN1ss) (**Table 5** **and Fig. S2 for diagrams of constructs**) proved that these genetically divergent bat-influenza virus vRNAs were highly interchangeable, in contrast to their very low compatibility with IAV and IBV. Interestingly, classical co-infection of the Bat09:mH1mN1 and Bat10:mH1mN1 viruses in MDCK cells readily generated reassortant progeny viruses with various genotypes, and some were apparently preferentially selected (e.g., Bat10:Bat09-NS reassortant, **Table S2**), demonstrating the merit of classic co-infection strategy in identification of gene constellations that may have certain advantages. Collectively the mini-genome replication, reverse genetics reassortment, and co-infection reassortment experiments strongly suggest that two divergent bat-influenza viruses readily reassort with each other, whereas they won't reassort with canonical IAVs in the natural setting.

**Table 5:** Rescue efficiency of reassortants between Bat09:mH1mN1ss and Bat10:mH1mH1ss.

	PB2	PB1	PA	NP	M	NS	HA	NA	Rescue*
**1**	Bat09	Bat09	Bat09	Bat09	Bat09	Bat09	H17ps-H1ss	N10ps-N1ss	**++++**
**2**	Bat10	Bat09	Bat09	Bat09	Bat09	Bat09	H17ps-H1ss	N10ps-N1ss	**+++**
**3**	Bat09	Bat10	Bat09	Bat09	Bat09	Bat09	H17ps-H1ss	N10ps-N1ss	**++**
**4**	Bat09	Bat09	Bat10	Bat09	Bat09	Bat09	H17ps-H1ss	N10ps-N1ss	**+**
**5**	Bat09	Bat09	Bat09	Bat10	Bat09	Bat09	H17ps-H1ss	N10ps-N1ss	**+++**
**6**	Bat09	Bat09	Bat09	Bat09	Bat10	Bat09	H17ps-H1ss	N10ps-N1ss	**++++**
**7**	Bat09	Bat09	Bat09	Bat09	Bat09	Bat10	H17ps-H1ss	N10ps-N1ss	**++++**
**8**	Bat09	Bat09	Bat09	Bat09	Bat09	Bat09	H18ps-H1ss	N10ps-N1ss	**++++**
**9**	Bat09	Bat09	Bat09	Bat09	Bat09	Bat09	H17ps-H1ss	N11ps-N1ss	**++++**
**10**	Bat09	Bat09	Bat09	Bat09	Bat09	Bat09	H18ps-H1ss	N11ps-N1ss	**+++**
**11**	Bat10	Bat10	Bat10	Bat10	Bat10	Bat10	H18ps-H1ss	N11ps-N1ss	**++++**
**12**	Bat09	Bat10	Bat10	Bat10	Bat10	Bat10	H18ps-H1ss	N11ps-N1ss	**+**
**13**	Bat10	Bat09	Bat10	Bat10	Bat10	Bat10	H18ps-H1ss	N11ps-N1ss	**+++**
**14**	Bat10	Bat10	Bat09	Bat10	Bat10	Bat10	H18ps-H1ss	N11ps-N1ss	**++**
**15**	Bat10	Bat10	Bat10	Bat09	Bat10	Bat10	H18ps-H1ss	N11ps-N1ss	**+++**
**16**	Bat10	Bat10	Bat10	Bat10	Bat09	Bat10	H18ps-H1ss	N11ps-N1ss	**+++**
**17**	Bat10	Bat10	Bat10	Bat10	Bat10	Bat09	H18ps-H1ss	N11ps-N1ss	**++++**
**18**	Bat10	Bat10	Bat10	Bat10	Bat10	Bat10	H17ps-H1ss	N11ps-N1ss	**+++**
**19**	Bat10	Bat10	Bat10	Bat10	Bat10	Bat10	H18ps-H1ss	N10ps-N1ss	**+++**
**20**	Bat10	Bat10	Bat10	Bat10	Bat10	Bat10	H17ps-H1ss	N10ps-N1ss	**+++**

*Rescue efficiency definition described in Table 1.

## Discussion

The generation of synthetic modified bat-influenza viruses (e.g., Bat09:mH1mN1) that grow to high titers in commonly used influenza virus culture substrates and mice is an important step toward understanding these novel bat-influenza viruses. The rescue of Bat09:mH1mN1 and Bat09:mH3mN2 viruses demonstrates that the putative vRNAs of Bat09 function efficiently together and are probably derived from either one virus, or a group of compatible viruses, whose PB2, PB1, PA, NP, M, and NS proteins efficiently replicate and package vRNAs in host cells commonly used to culture influenza viruses (**Fig. 1**). Importantly, the data also shows that the bat-influenza HA and NA were the sole determinants inhibiting Bat09 virus rescue, and that the terminal regions of HA and NA of bat-influenza viruses selected for our constructs contain cis-acting vRNA packaging signals. Although wild type bat-influenza virus (Bat09) couldn't be propagated in the human, canine, mink, avian, porcine or bat cell lines we tested, consistent with Tong et al. [3], it is likely that the bat-influenza virus can infect some other cell cultures from other species and/or tissues, especially cells derived from appropriate bat species.

Our Bat09:mH1mN1 studies provide other unique insights, which can't be gleaned from non-infectious assays. For instance, non-infectious assays (interferon-β reporter assay, **Fig. 4A**) showed the Bat09 NS1 carboxy-terminal truncationss (NS1-128 and NS1-73) were similar to the truncated PR8 NS1 (NS1-126 and NS1-73), which largely lost the ability to inhibit the host interferon response. However, mouse experiments with the replicative bat-influenza viruses revealed that the truncation of Bat09 NS1 had minimal effects on the viral pathogenesis compared to the truncation of PR8 NS1 (**Fig. 5**). Differences in the attenuating impact observed in the PR8-NS1 and the Bat09-NS1 truncated viruses suggests that Bat09 has novel molecular mechanisms that have evolved in the amino terminal portion of NS1 and/or other internal protein vRNAs to antagonize/evade the host innate immune response.

The PB2 of IAV plays important roles in replication, species specificity, transmission, and pathogenesis [23]–[29]. Our analysis of bat-influenza PB2 demonstrated that it is also a virulence determinant and as anticipated conversion of mammalian-signature residues at position 701 to avian-signature (N701D) attenuated the virus, and the E158G substitution [23] enhanced virulence. PB2-627 is one of the most studied positions differentiating avian viruses (glutamic acid) and mammalian viruses (lysine) [24], [27]. Intriguingly, the bat-influenza PB2 has a serine at position 627, which is unlike mammalian or avian IAVs. Our data show that PB2-627S has intermediate polymerase activity compared to PB2-627E and PB2-627K in mammalian cells, suggesting an alternative evolutionary pathway that avian influenza viruses may be able to take for mammalian adaptation.

Reassortment of the segmented genomes of *Orthomyxoviruses is* a powerful evolutionary mechanism that is central to the success of these pathogens. Viruses within a Genus readily reassort upon co-infection of a single host cell (e.g., avian and swine IAV); whereas, viruses from a different Genus (e.g., IAV and IBV) don't reassort. The factors important for generation of reassortant progeny from two parental influenza viruses include: recognition and replication of vRNAs by parental virus RdRp, protein-protein interaction/compatibility (e.g, heterotrimeric RdRp), and vRNA-protein interactions needed for virion morphogenesis. The RNA transcription/replication promoter of each influenza vRNA segment is formed by base pairing of highly conserved nucleotides at the 5′ and 3′ termini, which form a partially double-stranded structure. The IAV Genus has specific nucleotide variations within the termini that distinguish it from IBV. The termini of bat-influenza vRNAs also show conserved 5′ and 3′ complementarity; however, they also have distinct nucleotide variation. Therefore, we used mini-genome replication studies to analyze promoter recognition and RdRp activity of various combinations of the PB1, PB2, PA subunits in combination with various NPs from IAV, IBV, or bat-influenza. The data show that the wild type RdRp most efficiently replicate their cognate vRNAs, and that both IAV and IBV RdRp have 50–60% reduction in activity with the bat-influenza mini-genome. Many PB1, PB2, PA combinations between bat-influenza and IAV/IBV dramatically reduce activity, which demonstrates protein-protein incompatibility between the RdRp subunits. Interestingly, the bat-influenza NP and IAV NP were completely compatible in the mini-genome assay, however NP reassortant viruses could not be generated (**Table 2** and **Table 3**) suggesting that the incompatibility of NPs may also involve complicated protein-vRNA interactions.

IAVs of various subtypes can infect and reassort in bat cell lines [30], [31], providing a permissive environment for them to reassort with bat-influenza viruses. However, our reassortant analysis indicates that while two divergent bat-influenzas readily reassort, bat-influenza and IAVs don't easily reassort in co-infection experiments. Reverse genetics reassortment studies showed the PB2, PB1, PA, NP, and NS vRNAs of bat-influenza don't efficiently reassort with the IAV or IBV, and provide many additional tantalizing results. For example, reassortants were not rescued from relatively compatible RdRp combinations in the mini-genome assay (e.g. Bat-PB2/PR8-PB1/PR8-PA, **Fig. 8A**) and demonstrate that divergent Bat09 and Bat10 can efficiently reassort with each other (**Table 5**). The M segment is the most highly conserved gene among influenza A and B viruses. We found that the PR8-M segment could substitute for the Bat09-M segment (**Table 2**), indicating that the M vRNAs/protein(s) of PR8 and Bat09 have enough conservation in both cis-acting packaging signals and functional domains of the proteins (M1/M2) to enable the replication of the modified Bat09 virus. In contrast, putative packaging signal swapping of the NP and NS segments didn't overcome reassortment defects suggesting that incompatibility at the protein-protein or protein-vRNA level is likely to be a critical factor inhibiting reassortment between the bat-influenza and other influenza viruses. Alternatively, one could argue that that since the vRNA packaging signals of bat-influenza NP and NS segments have not been delineated, the putative packaging regions incorporated in the Batps-PR8 constructs may not be sufficient for packaging the modified vRNAs. However, the well-defined PR8 packaging signals incorporated in our modified gene segments should be sufficient to package the corresponding bat-influenza NP and NS vRNAs (PR8ps-Bat-NP and PR8ps-Bat-NS, **Fig. S2D**) in the PR8 backbone. The failure to rescue the PR8ps-Bat NP or NS viruses, as well as the PR8:Bat09-M reassortant virus, strongly suggests protein-protein or protein-vRNA level incompatibility and provides a unique opportunity to better understand the functional domains of these proteins through characterizing chimeric/mosaic proteins containing motifs/domains from both viruses.

Another caveat with our bat-influenza reassortment experiments is the focus on interactions with the laboratory adapted PR8 virus, which was chosen primarily due to biosafety concerns. Reassortment between the Bat09:mH1mN1 virus and other IAVs, particularly avian viruses (e.g., H5N1, H7N9) that appear to be more compatible in the mini-genome assay (**Fig. 8**), are needed to fully assess reassortment potential of bat-influenza. However, based on our results from the NP reassortment and the Bat-PB2/PR8-PB1/PR8-PA reassortment experiments (**Table 1** and **Table 2**), the likelihood of rescuing a reassortant with RdRp components from both Bat and IAVs is very low. Finally, since the HAs and NAs of the bat influenza viruses can't be used to rescue viruses using contemporary influenza virus host substrates, we were not able to fully assess the ability of the HA or NA to reassort with other influenza viruses (limited assessment provided in **Table 4**). However, the known bat influenza viruses (Bat09, Bat10) could pose a pandemic threat if their HA and NA acquire mutations that impart binding to canonical influenza virus receptors and rescuing the NA for neuraminidase activity, or acquisition of binding and entry through alternative human cell surface receptors.

Collectively, our experiments suggest that the bat-influenza virus is unlikely to reassort with an IAV or IBV and spread to other species even if they were to infect the same host cell. The restriction on reassortment appears to result from multiple levels of incompatibility (RNA-RNA, RNA-protein, and/or protein-protein) that are either additive or synergistic. Consequently, our data suggest that due to the extremely limited ability of genetic information exchange between bat-influenza and IAV or IBV, the International Committee on Taxonomy of Viruses could consider classifying these two bat-influenza virus lineages as a new Genus or Species within the *Orthomyxoviridae*.

This study also demonstrated the power of synthetic genomics in rapid characterization and risk assessment of an emerging virus, even when the virus itself is not readily cultured. The synthetic genomics/reverse genetics strategy employed provides an infinite supply of wild type bat-influenza particles that can be used to identify permissive cells or animals. The availability of our modified bat-influenza virus, opens many other avenues of investigation and discovery, including, for instance, to gain a better understanding of cis-acting signals in the vRNAs that are important in bat-influenza transcription, replication, packaging/particle morphogenesis, and to use forward genetics to elucidate viral protein-protein and/or viral protein-host protein interactions. Finally, continued study of bat-influenza viruses and integration of data from other contemporary influenza viruses is important in the elucidation of the evolutionary history of influenza viruses.

## Materials and Methods

### Biosafety and ethics statement

The study was reviewed and approved by the Institutional Biosafety Committee at Kansas State University (protocol #903), and by the institutional biosafety committee at the J. Craig Venter Institute (protocol # 3414). We conducted the initial studies using PR8 gene fragments to generate the modified bat-influenza viruses and to test the reassortment potential because PR8 is a widely used lab/mouse adapted BSL2 virus that poses very low risk to humans or livestock. Subsequently, TX98 H3N2 genes were used in a few experiments because this is a BSL2 swine virus, which we have used previously and the viruses generated were considered low risk.

The animal studies were performed in strict accordance with the recommendations in the Guide for the Care and Use of Laboratory Animals of the National Institutes of Health. The animal protocol (protocol #3339) was reviewed and approved by the Institutional Animal Care and Use Committee at Kansas State University. All animal studies were performed in a Biosafety Level 3 facility located at the Biosecurity Research Institute at Kansas State University under the approved protocol #3339 following the American Veterinary Medicine Association guidelines on euthanasia. For virus inoculation, each mouse was anesthetized by inhaling 4% isoflurane. Mice were euthanized if more than 25% of weight was lost after virus inoculation. Euthanasia of mice was conducted by inhaling 4% isoflurane followed by cardiac puncture and cervical dislocation. No survival surgery was performed, and all efforts were made to minimize suffering.

### Cells

Human embryonic kidney 293T (HEK-293T) cells, mouse rectum epithelial carcinoma (CMT-93) cells, and African green monkey kidney (Vero) cells were maintained in Dulbecco's modified Eagle's medium (DMEM) supplemented with 10% fetal bovine serum (FBS). Madin-Darby canine kidney (MDCK) cells were maintained in minimum essential medium (MEM) supplemented with 5% FBS. Human lung epithelial (A549) cells, bat lung epithelial (Tb1Lu) cells, mink lung epithelial (Mv1Lu) cells and swine testis (ST) cells were maintained in MEM supplemented with 10% FBS. Human lung epithelial (Calu-3) cells were maintained in MEM supplemented with 10% FBS, 1% nonessential amino acids, and 1 mM sodium pyruvate.

### Complete genome synthesis and plasmid construction

Nucleotide sequences of the eight gene segments of A/little yellow-shouldered bat/Guatemala/164/2009 (H10N17) (Bat09) were retrieved from the GenBank database. A total of 472 oligonucleotides of 56–60 bases in length were designed for enzymatic assembly of the eight segments. The assembly and error correction processes were performed as recently described [32], [33], modified with increased time at all extension steps (from 72°C for 1 min to 72°C for 2 min) for efficient assembly of the polymerase segments. The synthesized segments (**Fig. S1**) were cloned into the modified bidirectional influenza reverse genetics vectors pBZ66A12 [34] using the recombination-based method [35] and transformed into Stella competent *E. coli* cells (Clontech). Colonies were selected and sequenced. The appropriate clones for each segment were propagated for plasmid preparation and verified by sequencing. The resulting plasmids are pBZ146A1 (PB2), pBZ147A11 (PB1), pBZ148A20 (PA), pBZ149A30 (HA), pBZ150A31 (NP), pBZ151A36 (NA), pBZ152A42 (M) and pBZ153A45 (NS). The whole process only took seven days to complete. The plasmids containing Bat09 PB2 mutations were constructed by site-directed mutagenesis using the pBZ146A1 as template. The NS1 truncation constructs were generated by Gibson assembly and details of the truncations are diagramed in **Fig. S2C**. The modified (m) Bat09 HA and NA (mH1, mN1, mH1ss, and mN1ss, see **Fig. S2A, S2B** for diagrams, and **Fig. S4** for sequence alignment) were synthetized by Gibson assembly from oligonucleotides. Silent substitutions (ss) were introduced to disrupt the putative packaging signals in the PR8 HA and NA terminal coding regions. The mH1ss and mN1ss are thus more appropriate than the mH1 and mN1 to assess the HA and NA packaging signal compatibility between Bat09 and PR8. The Batps-PR8-NP, PR8ps-Bat-NP, Batps-PR8-NS, and PR8ps-Bat-NP constructs were constructed similarly and diagramed in **Fig. S2D**. As a comparison of the speed of different synthesis strategies, the eight gene segments of A/flat-faced bat/Peru/033/2010 (H18N11) (Bat10) were synthesized by Genewiz (NJ, USA) in the vector plasmid of pUC57 based on the GenBank database and subcloned into pHW2000 vector. The resulting plasmids (pHW-H18-PB2, pHW-H18-PB1, pHW-H18-PA, pHW-H18-HA, pHW-H18-NP, pHW-H18-NA, pHW-H18-M and pHW-H18-NS) were confirmed by sequencing. The whole process took more than one month. The PB2, PB1, PA and NP genes were also subcloned into the pDZ vector for use in the mini-genome assay. Diagrams of the mutant or modified genes of Bat09 and Bat10 are described in **Fig. S2**. The pPol1-NS-Luc reporters used in the mini-genome polymerase activity assay were described in **Fig. S2E**. Sequences of all constructs used in this study were confirmed to ensure absence of unwanted mutations and the GenBank accession numbers are KM203345-KM203356.

### Virus rescue

Briefly, 0.6 µg of plasmid for each gene segment was mixed and incubated with 15 µl of Mirus TranIT-LT1 (Mirus Bio, Madison, WI) at 20°C for 20 min. The transfection mixture was transferred to 90% confluent 293T/MDCK cell monolayers in a 35-mm tissue culture dish and incubated at 37°C with 5% CO_2_ for 8 h. The transfection supernatant was replaced with 3 ml of Opti-Mem I medium (Life Technologies) supplemented with 0.3% bovine serum albumin (BSA) fraction V (Life Technologies), 3 µg/ml tosylsulfonyl phenylalanyl chloromethyl ketone (TPCK)-trypsin (Worthington, Lakewood, NJ), and 1% antibiotic-antimycotic (Life Technologies). Three days post-transfection, culture supernatant (passage 0, P0) was collected and 0.5 ml of that was inoculated into MDCK cells in 6-well plates at 37°C. Supernatant (P1) was collected at 4 days post-inoculation (dpi), or when severe cytopathic effect (CPE) was observed. The P1 supernatant was further passaged blindly for two passage before determined to be negative for rescue. Titers of the viruses used in this study were determined by TCID_50_ assay in MDCK cells.

Rescue efficiency definition. **Very easy (++++)**: P0 viral titer 10^6^–10^8^ TCID_50_/ml, or severe CPE observed in P1 within 1 dpi; **Moderate (+++)**: P0 titer 10^4^–10^6^ TCID_50_/ml, or obvious CPE observed in P1 within 2 dpi; **Difficult (++)**: P0 titer 10^2^–10^4^ TCID_50_/ml, or weak CPE observed in P1 within 4 dpi; **Very difficult (+)**: P0 titer lower than 10^2^ TCID_50_/ml, or CPE not observed until P2/P3; **Negative (Neg)**: rescue failed, no CPE observed through passage 3.

Various transfection conditions including different transfection reagents, temperatures, and incubation time before supernatant collection were attempted to rescue the wild type Bat09 virus and the reassortants between Bat09 and PR8. However, none of them generated any positive rescue results if they were negative under standard rescue condition described above. Bat09 transfection supernatants were also transferred to various cells (MDCK, mink lung Mv1-Lu, swine testis, Vero, A549 cells, Calu-3, bat lung epithelial Tb1Lu) and embryonated chicken eggs and passaged at least three times. The real-time RT-PCR assays targeting Bat09 and PR8 M genes were used to confirm negative results (primers and probes are possible upon request).

### Electron microscopy

To determine whether virus particles of Bat09 and other viruses can be produced by reverse genetics system, a total of thirty-five ml of transfected 293T cell supernatants for each virus were collected at 48 hours post transfection and centrifuged at 8000 rpm for 20 minutes to remove the cell debris. Then the clear supernatant was loaded on 30% (w/v) sucrose in centrifuge tubes and was concentrated at 27,000 rpm (Optima LE-80K ultracentrifuge, Beckman Coulter) for 2 hours. The virus pellets was dissolved in 100 µl of water and the viral particles were fixed by incubating with 0.2% paraformaldehyde at 37°C for 48 hours. The fixed particles were dipped on a 200 mesh copper grid and the grid was dried and stained with negative staining before observation under an electron microscope.

### Virus replication *in vitro* and *in ovo*

MDCK monolayers in 12-well plates were washed twice with PBS, and then 2 ml of virus growth medium (VGM) was added to each well. The cells were inoculated at a multiplicity of infection (MOI) of 0.01 TCID_50_/cell with the Bat09:mH1mN1 virus or PR8 virus (Bat09:mH3mN2 virus or TX98 virus) and incubated at 37°C. Supernatants were collected at 1, 2, and 3 days post inoculation (dpi). Inoculations of Calu-3 cells were performed similarly, except that an MOI of 0.02 TCID_50_/cell was used for the following viruses: Bat09:mH1mN1ss, Bat09:mH1mN1ss-NS1-73, Bat09:mH1mN1ss-NS1-128, PR8, PR8-NS1-73, and PR8-NS1-126. The VGM used for MDCK cells was EMEM supplemented with 0.15% BSA fraction V, 2 µg/ml TPCK-trypsin, and 1% antibiotic-antimycotic, and the VGM used for Calu-3 cells was EMEM supplemented with 0.3% BSA fraction V, 1 µg/ml TPCK-trypsin, and 1% antibiotic-antimycotic. All virus titers were determined by TCID_50_ assay using MDCK cells.

Six of 10-day-old embryonated chicken eggs were inoculated with Bat09:mH1mN1 or PR8 at 10^3^ TCID_50_/egg. After 2 days incubation at 35°C, allantoic fluid was collected from each egg and titrated individually. The 4 eggs with the highest titers in each virus group was used to calculate the average titer and generate the graph in **Fig. 1E**.

### Next generation sequencing and analysis

A modified Multi-segment RT-PCR [35], [36] was used to amplify influenza-specific segments. The only modification to the procedure was the primers used for amplification were changed to match bat influenza termini. The oligonucleotide primers used were Uni12/Inf-5G (5′-GGGGGGAGCAGAAGCAGG-3′) and Uni13/Inf-1 (5′-CGGGTTATTAGTAGAAACAAGG-3′). The M-RTPCR amplicons were used for Illumina Miseq library construction via Nextera DNA sample prep kit (Illumina, Inc.) and sequenced using the Illumina MiSeq (Illumina, Inc.) according to manufacturer's instructions. SNP variations were identified using custom software that applies statistical tests to minimize false positive SNP calls that could be caused by the types of sequence-specific errors that may occur in Illumina reads identified and described in Nakamura, et al. [37]. To overcome this problem, the protocol requires observing the same SNP, at a statistically significant level, in both sequencing directions. Once a minimum minor allele frequency threshold and significance level are established by the user, the number of minor allele observations and major allele observations in each direction and the minimum minor allele frequency threshold are used to calculate a p-value based on the binomial distribution cumulative probability, and if the p-values calculated in each of the two sequencing directions are both less than the Bonferroni-corrected significance level, then the SNP call is accepted. For our analyses, we used a significance level of 0.05 (Bonferroni-corrected for tests in each direction to 0.025), and a minimum minor allele frequency threshold of 10% of the read population.

### Interferon-β reporter assay

To measure the IFN-antagonist function of NS1, a luciferase-based, Sendai virus-mediated IFN-β promoter activation assay was conducted as previously described [16]. Briefly, 293T cells in 24-well plates were transfected with empty vector (200 ng) or increasing amounts of wild type (WT) or carboxyl terminal truncated NS1 from Bat09 and PR8 (2 ng, 10 ng, and 50 ng of NS1 expression plasmids supplemented with 198 ng, 190 ng, and 150 ng of empty vector, respectively). Also co-transfected were 200 ng of an IFN-β-promoter-luciferase reporter plasmid (pIFNβ-Luc) and 20 ng of a plasmid constitutively expressing Renilla luciferase (pRL-TK from Promega). At 18 hours post transfection, cells were infected with Sendai virus to induce the IFN-β promoter. A dual-luciferase assay was performed at 18 hour post virus inoculation, and firefly luciferase was normalized to Renilla luciferase activity. The relative luciferase activity of the group with empty vector was set as 100%, and the other groups were presented relative to that.

### Interferon bioassay with VSV-luciferase virus

As previously described for the VSV-GFP virus mediated interferon bioassay [22], in the VSV-Luciferase virus mediated bioassay, A549 cells were inoculated with one of the wild type or NS1 truncated viruses at an MOI of 4 TCID_50_/cell, or were mock-inoculated; supernatants were then collected at 24 hpi. Supernatants were treated with UV irradiation to inactivate viruses and were then transferred to naïve A549 cells. Following 24 h of incubation at 37°C, supernatants were removed, and the cells were inoculated with VSV-Luciferase virus [38], at an MOI of 2 TCID_50_/cell. The firefly luciferase expression in the cells was measured using the Luciferase Assay System (Promega) at 4 hpi with VSV-Luciferase.

### Mini-genome polymerase activity assay

The luciferase-mediated mini-genome polymerase activity assay was performed as previously described, using a PolI-driven reporter plasmid and pDZ-based PB2, PB1, PA, and NP bidirectional expression plasmids [21], [35]. To determine the effects of PB2 mutations on polymerase activity (**Fig. 7**) 293T cells were co-transfected with 0.2 µg each of the PB2 (WT or mutant), PB1, PA, NP, and a pPol1-FluA-NS-Luc (firefly luciferase flanked by A/New York/1682/2009 [23]). As a control for transfection efficiency, 0.02 µg of the Renilla luciferase plasmid pRL-TK (Promega) was also co-transfected. After 18 hours of incubation at 33°C, 37°C, and 39°C, luciferase production was assayed using the dual-luciferase reporter assay system (Promega) according to the manufacturer's instructions. Firefly luciferase expression was normalized to Renilla luciferase expression (relative activity). The relative activity of the PB2-WT was set as 1 fold, and the relative activities of the PB2 mutants were presented relative to that (**Fig. 7**).

To test the compatibility between RNPs (PB2, PB1, PA, and NP) and viral RNA promoters from bat-influenza virus (Bat09) (**Fig. S3**), IAV (A/PR/8/1934), and IBV (B/Russia/1969), 293T cells were co-transfected with 0.2 µg each of the PB2, PB1, PA, NP, and a pPol1-NS-Luc reporter plasmid, followed by incubation at 37°C for 18 hours. Three reporters were used in this study, including pPolI-Bat-NS-Luc (firefly luciferase flanked by Bat09 NS non-coding regions), pPol1-FluA-NS-Luc, and pPolI-FluB-NS-Luc (firefly luciferase flanked by B/Russia/1969 NS non-coding regions) (**Fig. S2D**). For each combination of RNP and pPolI-NS-Luc reporter (from Bat09, A, or B Type), three independent replicates were conducted. For each RNP, the luciferase activity with the reporter from the same virus (e.g., Bat-RNP and pPol1-Bat-NS-Luc) was set at 100%, and the activities with the other two reporters (e.g., pPol1-FluA-NS-Luc and pPol1-FluB-NS-Luc) were presented relative to that (**Fig. S3**).

The PB2, PB1, PA, and NP compatibility between Bat09 and the following influenza viruses was examined in the study (**Fig. 8**): A/PR/8/1934 (lab adapted human H1N1), A/Ann Arbor/6/1960 (human H2N2), A/New York/238/2005 (human H3N2); A/New York/1692/2009 (human H1N1 seasonal), A/New York/1682/2009 (human H1N1 pandemic), A/canine/New York/6977983/2010 (canine H3N8), A/turkey/Ontario/7732/1966 (avian H5N9), A/Hong Kong/213/2003 (avian H5N1), A/Anhui/1/2013 (human H7N9), B/Russia/1969 (lab adapted human IBV), and A/flat-faced bat/Peru/033/2010 (bat H18N11). For the compatibility test between Bat09 and IAVs (**Fig. 8A–I**), 293T cells were co-transfected with 0.2 µg each of the PB2, PB1, PA, NP (from Bat09 or IAV), 0.1 µg of pPolI-Bat-NS-Luc plasmid and 0.1 µg of pPolI-FluA-NS-Luc. For compatibility test between Bat09 and IBV (**Fig. 8J**), 293T cells were co-transfected with 0.2 µg each of the PB2, PB1, PA, NP (from Bat09 or B/Russia/1969), 0.1 µg of pPolI-Bat-NS-Luc plasmid and 0.1 µg of pPolI-FluB-NS-Luc. For compatibility test between Bat09 and Bat10 (**Fig. 8K**), 0.2 µg each of the PB2, PB1, PA, NP (from Bat09 or Bat10), and pPolI-Bat-NS-Luc plasmids were used (The NS non-coding regions of Bat09 and Bat10 have the same sequence). Renilla luciferase was also co-transfected and dual-luciferase reporter assay system was used. For each combination of PB2, PB1, PA, and NP (from Bat09 or another influenza virus), three independent replicates were conducted at 37°C, the luciferase activity of the all-Bat09-combination (Bat09-PB2/Bat09-PB1/Bat09-PA/Bat09-NP) was set at 100%, and the activities of other 15 combinations were presented relative to that.

### Pathogenicity of PR8, modified bat-influenza virus (Bat09:mH1mN1) and PB2 mutants

A total of 98 female BALB/c mice aged 6 to 7 weeks were randomly allocated to 7 groups (14 mice/group). Six mice were intranasally inoculated with 10^3^ TCID_50_ of each virus (Bat 09:mH1mN1, Bat09:mH1mN1-PB2-701D, Bat09:mH1mN1-PB2-627K701D, Bat09:mH1mN1-PB2-158G701D, Bat09:mH1mN1-PB2-158G, PR8, or MEM Mock) in 50 µL fresh MEM medium while under light anesthesia by inhalation of 4% isoflurane. To determine the virus replication in mouse lungs, three mice from each group were euthanized on both 3 and 5 day post-inoculation (dpi). Another 8 mice from each group were intranasally inoculated with 10^4^ TCID_50_ of viruses in 50 µL MEM medium; all eight mice were kept to monitor body weights and clinical signs. Weights were recorded daily and general health status was observed twice daily. After the onset of disease, the general health status was observed three times daily. Severely affected mice (i.e., more than 25% body weight loss) were euthanized immediately, and the remaining mice were euthanized on 14 dpi. All control mice were intranasally inoculated with 50 µL fresh MEM (mock group), three control mice were necropsied at 3 and 5 dpi, the remaining mice were kept until the end of the animal study.

During necropsy, the right part of the lung was frozen at −80°C for virus titration, and the left part of the lung was fixed in 10% formalin for histopathologic examination. For virus titration, the 10% lung homogenate was prepared in cold fresh MEM medium by using a Mini Bead Beater-8 (Biospec Products; 16 Bartlesville, OK). The homogenate was centrifuged at 6000 rpm for 5 minutes, and the supernatant was titrated by infecting MDCK cells in 96-well plates. For the histopathologic examination, lung tissues fixed in 10% phosphate-buffered formalin were processed routinely and stained with hematoxylin and eosin. The lungs were examined microscopically both for the percentage of the lung involved and for the histopathologic changes seen, including bronchiolar and alveolar epithelial necrosis, intraalveolar neutrophilic inflammation, peribronchiolar inflammation, and bronchiolar epithelial hyperplasia and atypia. For detection of virus NP antigens in lung sections on day 5 post infection, a rabbit anti-H1N1 (2009 flu pandemic) NP polyclonal antibody was used (Genscript, USA). A pathologist examined each slide in a blinded fashion.

### Pathogenicity of modified bat-influenza viruses (Bat09:mH1mN1ss) containing truncated NS1 genes

A total of 70 female BALB/c mice aged 6 to 7 weeks were randomly allocated to 5 groups (14 mice/group). To determine virus replication, six mice were intranasally inoculated with 10^4^ TCID_50_ of each virus (Bat09:mH1mN1ss-NS1-WT, Bat09:mH1N1ss-NS1-73, Bat09:mH1mN1ss-NS1-128, and PR8-NS1-126) in 50 µL MEM medium while under light anesthesia by inhalation of 4% isoflurane. Three mice from each group were killed on both 3 and 5 day post-inoculation (dpi). Another 8 mice from each group were intranasally inoculated with 10^5^ TCID_50_ of each virus in 50 µL MEM medium for morbidity and mortality comparison. All the other procedures are same with described previously.

### Pathogenicity of TX98 and modified bat influenza (Bat09:mH3mN2) viruses

A total of 42 female BALB/c mice aged 6 to 7 weeks were randomly allocated to 3 groups (14 mice/group). To investigate virus replication in mice, six mice from each group were intranasally inoculated with 3×10^4^ TCID_50_ of virus or mock-inoculated with 50 µL fresh MEM medium while under light anesthesia by inhalation of 4% isoflurane. Three of six inoculated mice from each group were euthanized at 3 and 5 day post-inoculation (dpi). To evaluate viral pathogenicity in mice, the remaining eight mice from each group were intranasally inoculated with 3×10^5^ TCID_50_ of virus (Bat09:mH3mN2, and TX98) in 50 µL fresh MEM medium or mock-inoculated with 50 µL fresh MEM medium. The mice were monitored body weights and general health status daily. After the onset of disease, the general health status was observed twice per day. Severely affected mice (i.e., more than 25% body weight loss) were humanly euthanized, and the remaining mice were euthanized and bloods were collected from each mouse to isolate serum for the HI assay at 14 dpi. Sample collection and analysis, and virus titration were performed as described above.

### Co-infection study for assessment of reassortment

To study the reassortment between Bat09:mH1mN1 and PR8 or Bat10:mH1mN1, confluent monolayer of MDCK cells in 6-well-plates were co-infected with both viruses (Bat09:mH1mN1 and PR8, or Bat09:mH1mN1 and Bat10:mH1mN1). Both modified Bat09:mH1mN1 and Bat10:mH1mN1 viruses showed similar replication kinetics in MDCK cells, whereas the PR8 replicated more efficiently than both modified viruses in MDCK cells. Therefore, for the co-infection study with PR8 and Bat09:mH1mN1 viruses, the cells were infected with the PR8 at MOI of 1 and with the Bat09:mH1mN1 at MOI of 4 (a ratio of both viruses is 1∶4). For the co-infection study with Bat09:mH1mN1 and Bat10:mH1mN1 viruses, the cells were infected with each virus at MOI of 1 (a ratio of both viruses is 1∶1). The co-infected MDCK cells were incubated at 37°C with 5% CO2 for 1 hour. After 1 hour of incubation, the supernatant was removed and the infected cells were washed with fresh MEM for 10 times. One mL of infection medium supplemented with 1 µg/mL TPCK-trypsin (Worthington, Lakewood, NJ) was added on cells. The supernatant containing progeny viruses was collected at 24 hours after inoculation. Plaque assays were performed in MDCK cells to select single virus from co-infected supernatants. The purified single virus (plaque) was amplified for further analysis. To identify the origin of each gene of the purified single virus, specific RT-PCR was used to differentiate internal genes from Bat09:mH1mN1, Bat10:mH1mN1 and PR8 viruses (primers for specific RT-PCR are available upon request). The surface HA and NA genes were differentiated by sequencing HA and NA non-coding regions (packaging signals) since three parental viruses contain identical HA and NA ORF sequences and different sequences in non-coding region (it is difficult to differentiate them by RT-PCR). For the RT-PCR, RNAs were extracted from each amplified single virus using a QIAamp Viral RNA Mini Kit (Qiagen). cDNA was synthesized by using the bat universal 12 primer (5′-AGCAGAAGCAGG-3′) for the samples from the co-infection study with Bat09:mH1mN1 and Bat10:mH1mN1 viruses, and by using a mixture of an IAV universal 12 primer (5′-AGCRAAAGCAGG-3′) and the bat universal 12 primer (5′-AGCAGAAGCAGG-3′) for the samples from the co-infection study with Bat09:mH1mN1 and PR8 viruses. If the origin of internal genes determined by the specific RT-PCR was inconclusive, sequencing was performed to confirm the results from specific RT-PCR (All sequence primers are available upon request).

### Statistical analysis

Luciferase activity, virus titers, and mouse weights were analyzed by using analysis of variance (ANOVA) in GraphPad Prism version 5.0 (GraphPad software Inc, CA). One-way ANOVA with Dunnett's multiple comparison test was used to determine the significance of the differences (P<0.05) among different groups. For simple comparisons, Student's *t* test was used to examine the significance of differences observed. Error bars represent standard deviation (±SD).

## Supporting Information

Figure S1 **Synthetic generation of the eight full-length genomic segments of A/little yellow-shouldered bat/Guatemala/164/2009 (Bat09).** The products were assembled from oligonucleotides and error corrected. L: 1 Kb Plus DNA ladder from Life Technologies.
(TIF)

Figure S2 **Diagrams of select constructs and viruses used in this study.** All constructs shown are in cDNA sense complementary to viral RNA. (**A**) Modified HA (mH1) and modified NA (mN1). To construct the mH1, PR8-HA coding region was flanked by the putative packaging regions from Bat09-HA and all ATG in the Bat09-HA 5′ packaging region were mutated. To construct the mN1, PR8-NA coding region was flanked by the putative packaging regions from Bat09-NA and all ATG in the Bat09-NA 5′ packaging region were mutated. Batps-B/NA was constructed similarly with the packaging regions from Bat09-NA and the coding region from B/Russia/1969-NA. (**B**) mH1ss was constructed by introducing 64 of silent substitutions into the coding region of mH1 to disrupt the remaining packaging signals in the PR8-HA coding region. mN1ss was constructed by introducing 90 of silent substitutions into the coding region of mN1 to disrupt the remaining packaging signals in the PR8-NA coding region. The mH1ss was referred as H17ps-H1ss and the mN1ss was referred as N10ps-N1ss in Table 5. The H18ps-H1ss and N11ps-N1ss have the HA and NA packaging regions from Bat10. (**C**) The wild type NS gene and the NS1 truncated NS gene from Bat09. NS1 truncated PR8-NS genes were constructed similarly. (**D**) Bat09 NP and NS coding regions flanked by putative cis-acting packaging regions from PR8 NP and NS. PR8 NP and NS coding regions flanked by putative cis-acting packaging regions from Bat NP and NS. (**E**) The pPol1-Bat-NS-Luc, pPol1-FluA-NS-Luc, and pPol1-FluB-NS-Luc reporter genes.
(TIF)

Figure S3 **Compatibility between RNPs and viral RNA promoters from different viruses.** Left, RNP from Bat09 and luciferase reporter flanked by NS non-coding regions from bat-influenza virus, IAV, and IBV. Middle, RNP from influenza A and the three luciferase reporters. Right, RNP from IBV and the three luciferase reporters. Within each group of RNP, * indicates P<0.05, compared to the vRNA reporter from the same type of virus as the RNP.
(TIF)

Figure S4 **Sequence alignment of PR8-HA, mH1, mH1ss and PR8-NA, mN1, mN1ss.**
(TIF)

Table S1 **Rescue result of reassortment between Bat09 and PR8.**
(TIF)

Table S2 **Co-infection results for reassortment between Bat09:mH1mN1 and Bat10:mH1mN1.**
(TIF)
