# Supplementary figures and images for: Characterization of Uncultivable Bat Influenza Virus Using a Replicative Synthetic Virus
Source: PLoS Pathog. 2014 Oct 2;10(10):e1004420. doi: 10.1371/journal.ppat.1004420 (PMC4183581; doi:10.1371/journal.ppat.1004420)

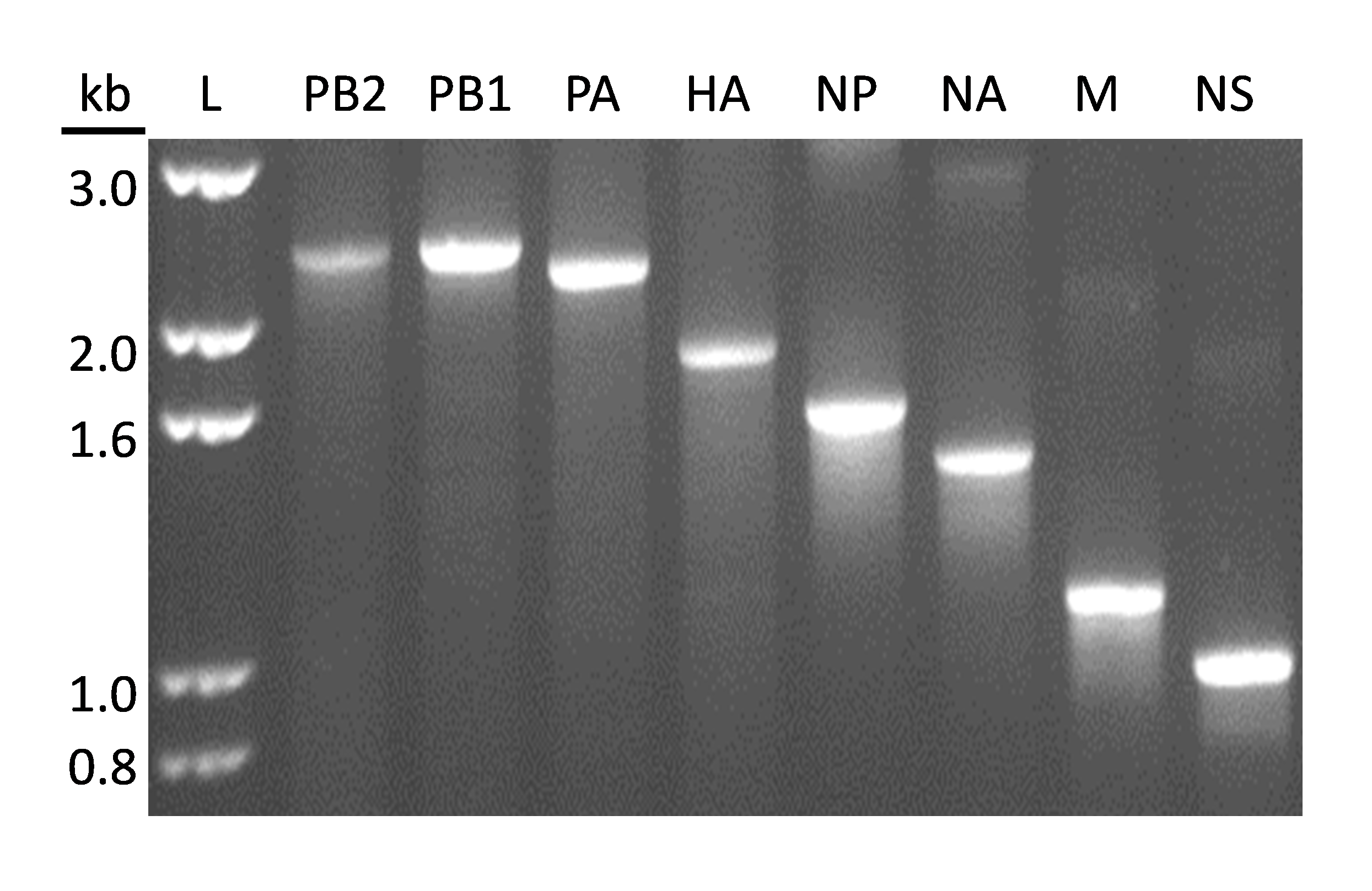

Supplement: Figure S1 — Synthetic generation of the eight full-length genomic segments of A/little yellow-shouldered bat/Guatemala/164/2009 (Bat09). The products were assembled from oligonucleotides and error corrected. L: 1 Kb Plus DNA ladder from Life Technologies. (TIF) [file ppat.1004420.s001.tif]

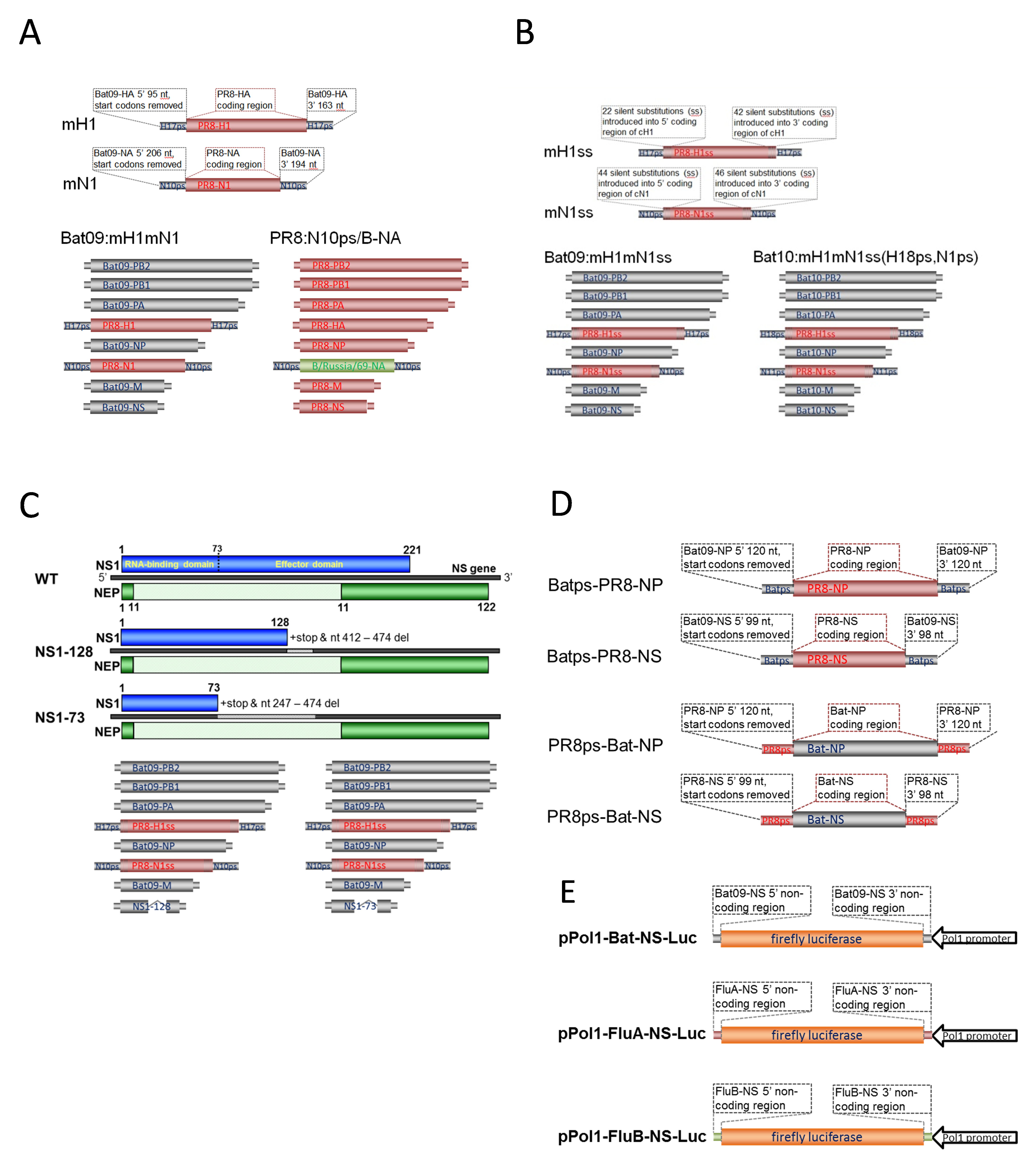

Supplement: Figure S2 — Diagrams of select constructs and viruses used in this study. All constructs shown are in cDNA sense complementary to viral RNA. (A) Modified HA (mH1) and modified NA (mN1). To construct the mH1, PR8-HA coding region was flanked by the putative packaging regions from Bat09-HA and all ATG in the Bat09-HA 5′ packaging region were mutated. To construct the mN1, PR8-NA coding region was flanked by the putative packaging regions from Bat09-NA and all ATG in the Bat09-NA 5′ packaging region were mutated. Batps-B/NA was constructed similarly with the packaging regions from Bat09-NA and the coding region from B/Russia/1969-NA. (B) mH1ss was constructed by introducing 64 of silent substitutions into the coding region of mH1 to disrupt the remaining packaging signals in the PR8-HA coding region. mN1ss was constructed by introducing 90 of silent substitutions into the coding region of mN1 to disrupt the remaining packaging signals in the PR8-NA coding region. The mH1ss was referred as H17ps-H1ss and the mN1ss was referred as N10ps-N1ss in Table 5. The H18ps-H1ss and N11ps-N1ss have the HA and NA packaging regions from Bat10. (C) The wild type NS gene and the NS1 truncated NS gene from Bat09. NS1 truncated PR8-NS genes were constructed similarly. (D) Bat09 NP and NS coding regions flanked by putative cis-acting packaging regions from PR8 NP and NS. PR8 NP and NS coding regions flanked by putative cis-acting packaging regions from Bat NP and NS. (E) The pPol1-Bat-NS-Luc, pPol1-FluA-NS-Luc, and pPol1-FluB-NS-Luc reporter genes. (TIF) [file ppat.1004420.s002.tif]

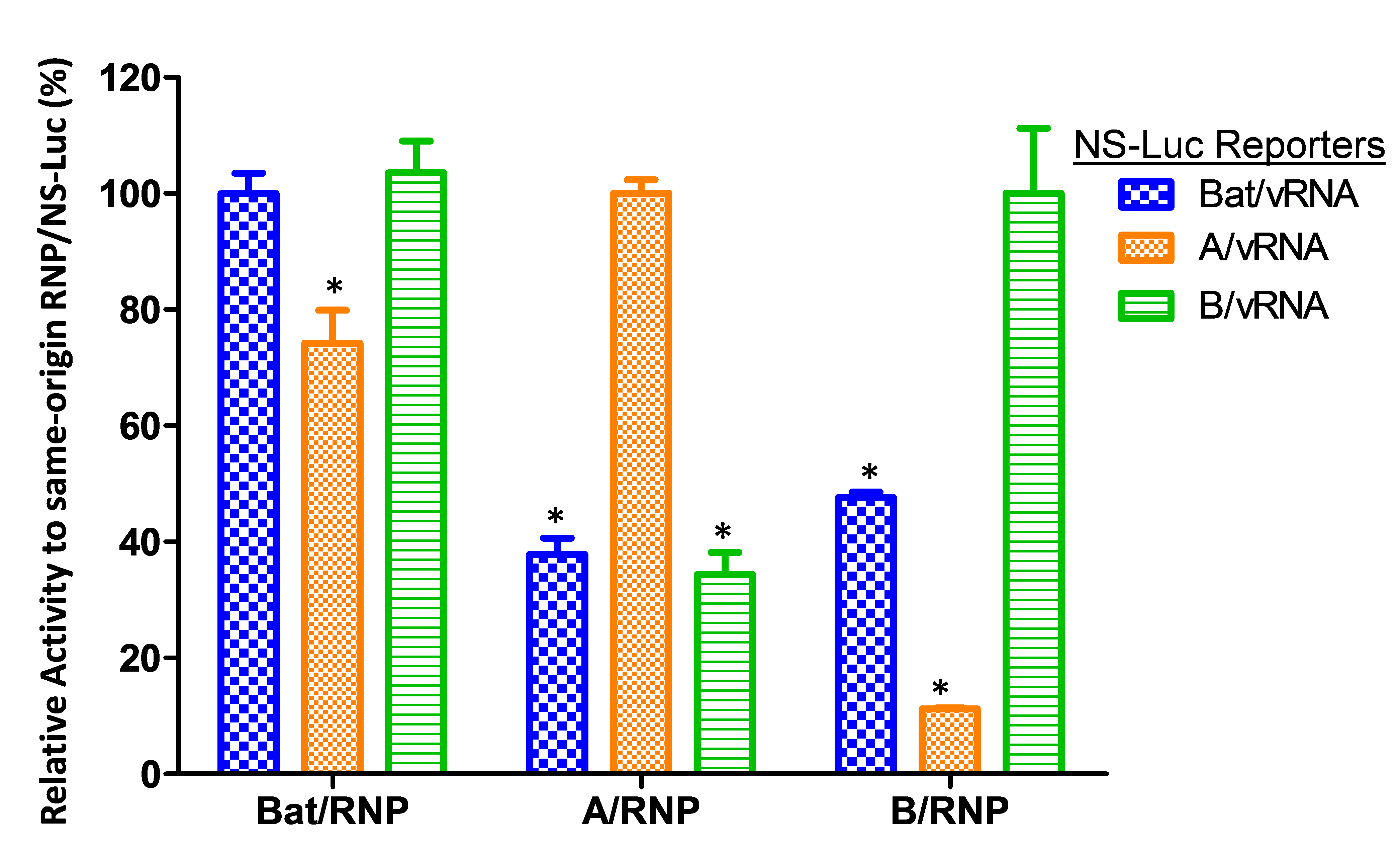

Supplement: Figure S3 — Compatibility between RNPs and viral RNA promoters from different viruses. Left, RNP from Bat09 and luciferase reporter flanked by NS non-coding regions from bat-influenza virus, IAV, and IBV. Middle, RNP from influenza A and the three luciferase reporters. Right, RNP from IBV and the three luciferase reporters. Within each group of RNP, * indicates P<0.05, compared to the vRNA reporter from the same type of virus as the RNP. (TIF) [file ppat.1004420.s003.tif]

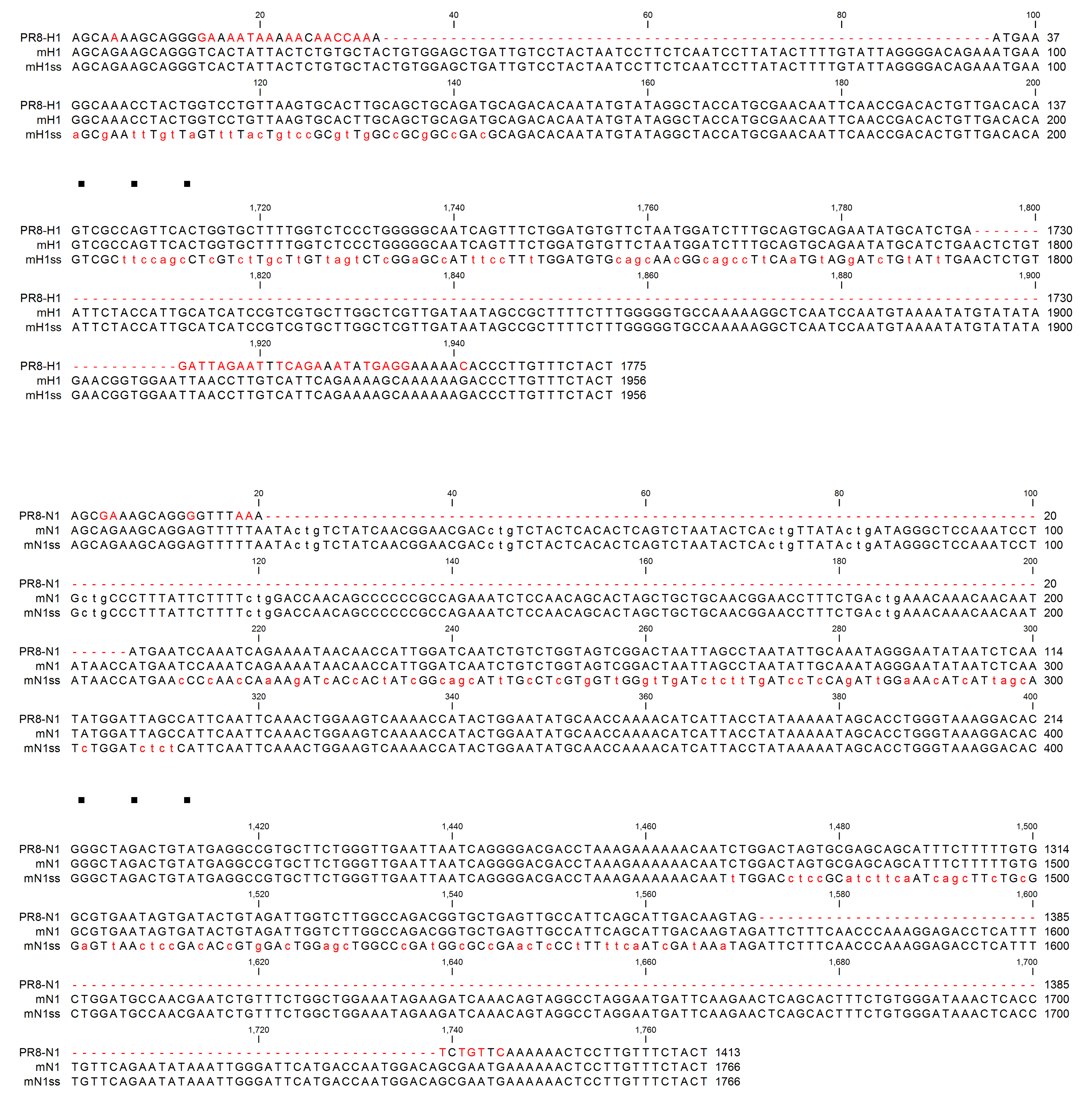

Supplement: Figure S4 — Sequence alignment of PR8-HA, mH1, mH1ss and PR8-NA, mN1, mN1ss. (TIF) [file ppat.1004420.s004.tif]

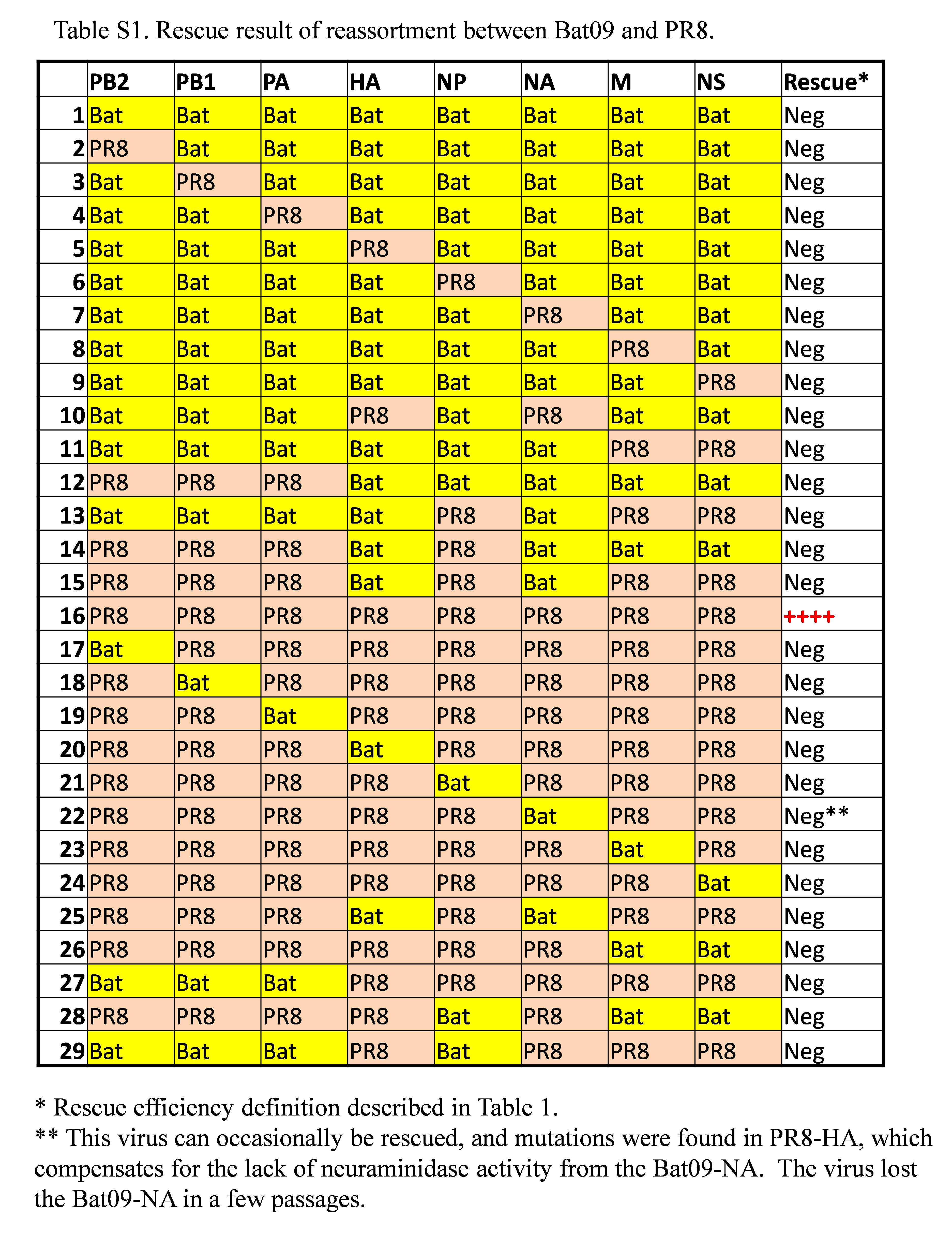

Supplement: Table S1 — Rescue result of reassortment between Bat09 and PR8. (TIF) [file ppat.1004420.s005.tif]

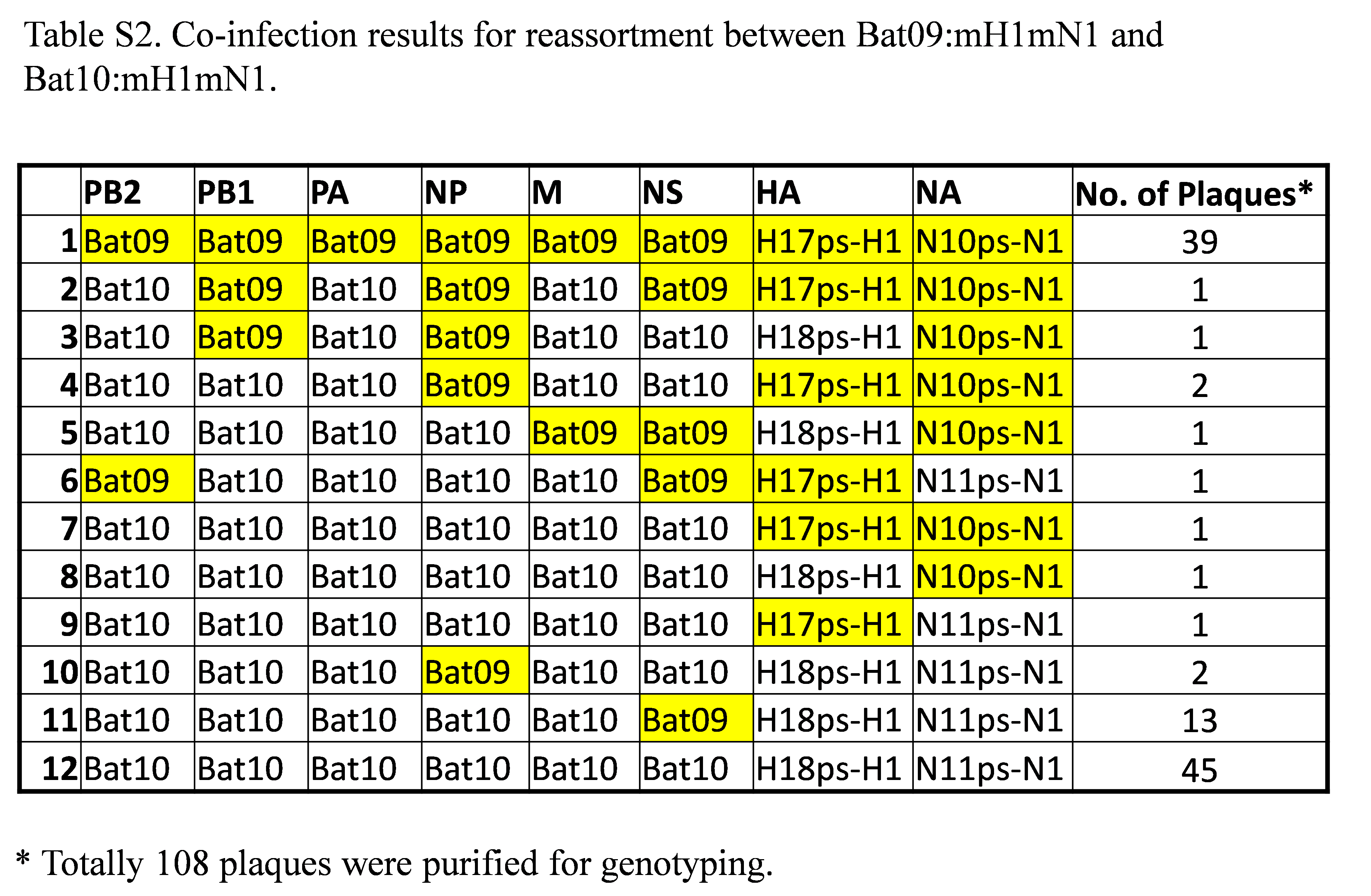

Supplement: Table S2 — Co-infection results for reassortment between Bat09:mH1mN1 and Bat10:mH1mN1. (TIF) [file ppat.1004420.s006.tif]
